# Transcriptional Regulation of Latency-Associated Transcripts (LATs) of Herpes Simplex Viruses

**DOI:** 10.7150/jca.40186

**Published:** 2020-03-05

**Authors:** Ying Zhang, Qiang Xin, Jun-Yi Zhang, Ying-Ying Wang, Jun-Ting Cheng, Wen-Qi Cai, Zi-Wen Han, Yang Zhou, Shu-Zhong Cui, Xiao-Chun Peng, Xian-Wang Wang, Zhaowu Ma, Ying Xiang, Xiu-Lan Su, Hong-Wu Xin

**Affiliations:** 1 The First School of Clinical Medicine, Health Science Center, Yangtze University, Nanhuan Road, Jingzhou, Hubei 434023, China.; 2 Laboratory of Oncology, Center for Molecular Medicine, School of Basic Medicine, Health Science Center, Yangtze University, 1 Nanhuan Road, Jingzhou, Hubei 434023, China; 3 Department of Biochemistry and Molecular Biology, School of Basic Medicine, Health Science Center, Yangtze University, Jingzhou, Hubei 434023, China.; 4 Clinical Medical Research Center, Affiliated Hospital of Inner Mongolia Medical University, Hohhot 010050, China.; 5 Department of Neural Surgery, People's Hospital of Dongsheng District of Erdos City, Erdos, Inner Mongolia, 017000, China.; 6 State Key Laboratory of Respiratory Disease, Affiliated Cancer Hospital Institute of Guangzhou Medical University, Guangzhou 510095, China.; 7 Department of Pathophysiology, School of Basic Medicine, Health Science Center, Yangtze University, Jingzhou, Hubei 434023, China.; 8 Department of Laboratory Medicine, School of Basic Medicine, Health Science Center, Yangtze University, 1 Nanhuan Road, Jingzhou, Hubei 434023, China.

**Keywords:** oncolytic Herpes Simplex Virus (oHSV), Latency-Associated Transcripts (LATs), transcription regulatory regions (TRRs), Transcription Regulatory Sequences (TRSs), Transcription Factors (TFs), latent neural infection

## Abstract

Herpes simplex viruses (HSVs) cause cold sores and genital herpes and can establish lifelong latent infection in neurons. An engineered oncolytic HSV (oHSV) has recently been approved to treat tumors in clinics. HSV latency-associated transcripts (LATs) are associated with the latent infection, but LAT transcriptional regulation was seldom reported. For a better treatment of HSV infection and tumors, here we sequenced the LAT encoding DNA and LAT transcription regulatory region of our recently isolated new strain HSV-1-LXMW and did comparative analysis of the sequences together with those of other four HSV-1 and two HSV-2 strains. Phylogenetic analysis of LATs revealed that HSV-1-LXMW is evolutionarily close to HSV-1-17 from MRC University, Glasgow, UK. For the first time, Using a weight matrix-based program Match and multi-sequences alignment of the 6 HSV strains, we identified HSV LAT transcription regulatory sequences that bind to 9 transcription factors: AP-1, C-REL, Comp1, E2F, Hairy, HFH-3, Kr, TCF11/MAFG, v-Myb. Interestingly, these transcription regulatory sequences and factors are either conserved or unique among LATs of HSV-1 and HSV-2, suggesting they are potentially functional. Furthermore, literature analysis found that the transcription factors v-myb and AP-1 family member JunD are functional in regulating HSV gene transcription, including LAT transcription. For the first time, we discovered seven novel transcription factors and their corresponding transcription regulatory sequences of HSV LATs. Based on our findings and other reports, we proposed potential mechanisms of the initiation and maintenance of HSV latent infection. Our findings may have significant implication in our understanding of HSV latency and engineering of better oncolytic HSVs.

## Introduction

Up to now, tumor treatments are still a big challenge. Although there are many methods to treat tumors 1-3, tumor recurrence rate is high and the metastasis is irreversible, resulting in poor clinical efficacy 4. Nowadays, there are mainly eight types of herpes virus, including herpes simplex virus type 1 and type 2 (HSV-1 and 2), varicella zoster virus (VZV), cytomegalovirus (CMV), Epstein-Barr virus (EBV), human herpes simplex virus types 6,7,8. Some herpes viruses cause tumors. For example, 27% of children with retinoblastoma are diagnosed with CMV infection 5. In addition, EBV is associated with nasopharyngeal carcinoma. However there is no direct evidence that herpes simplex virus causes tumors, so that HSV can be engineered as oncolytic HSV (oHSV) to combat cancer 6-9. In oHSVs, HSV can infect a variety of host cells to meet the needs of oncolytic virus therapy, and various forms of transgenic vectors have been developed for cancer therapy 10, 11. For example, the most advanced oHSVs T-Vec (Talimogene laherparepvec), G207, 1716, G47Δ and HF10 have been evaluated in clinical trials for their benefits in treating advanced cancers such as melanoma, glioma, head and neck cancer and breast cancer 12. Furthermore, a novel oHSV Ld0-GFP (which was derived from the oncolytic ICP0-null HSV) targeting hepatocellular carcinoma (HCC) has recently been reported 12. In 2016, the oHSV T-VEC has been approved for the treatment of melanoma 13,14.

Herpes viruses are classified into α, β and γ three genera 15. Among them γ herpes viruses include HSV-1 and 2 16, and are able to build lifelong latent infections in neuron 15. HSVs have a wide host range, and specific therapeutic agents (acyclovir). Initially, a HSV effectively infects epithelial cells or tissues (lytic infections) and then slowly spreads further around, stopping to form latent infections in the nucleus of a sensory neuron 17. HSVs express immediate early (IE or α), early (E or β) and late (L or γ) genes 18. IE genes encode five infected cell polypeptide (ICP) proteins, ICP0/RL2, ICP4/RS1, ICP22/US1, ICP27/UL54, and ICP47/US12 19. ICP0 plays an essential role in virus replication, cell growth and apoptosis 20, 21. ICP4 has multiple target sites recognized by CD8+ T cells and is a very important key regulator of early and late gene expression 22.

During latent infection of sensory ganglia in mammalian host 23, only the latency-associated transcripts (LATs) is the product of abundant expression of HSVs 24. LATs play a crucial role in establishing latency 25, maintaining latency 26, 27, reactivation of the virus from latency, and protection of neurons from apoptosis 28, 29. For understanding the function of LAT genes, we need to lean the structure of LATs and their transcriptional regulation in host cells.

**Figure 1:**
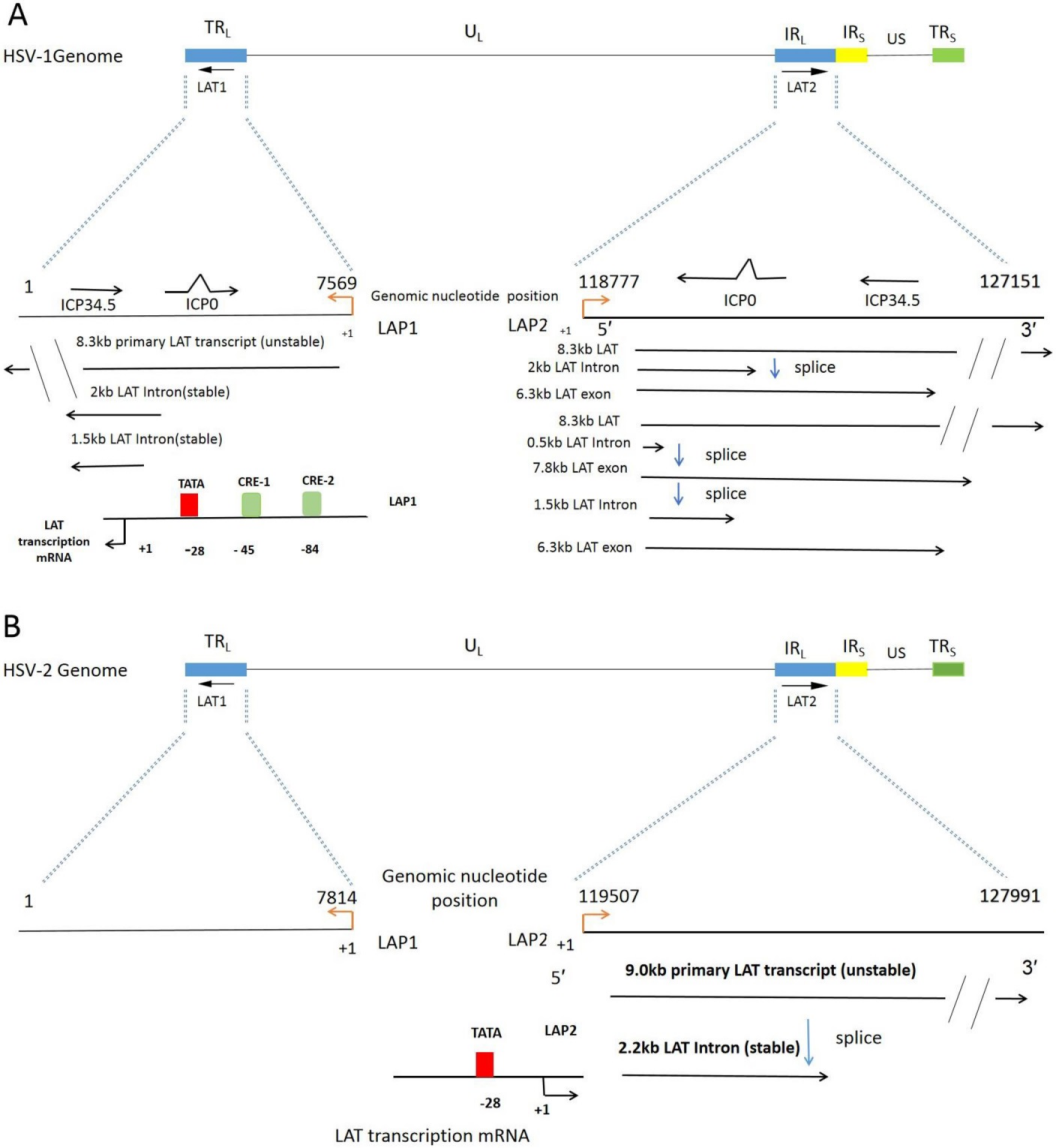
** The LAT and its LAT Twintron processing. A.** The linear HSV-1 (strain 17) genome contains with the Unique Long (U_L_) and Unique Short (U_S_) regions flanked by the terminal and internal Repeats (TR_L_, IR_L_, IR_S_ and TR_S_). LAT genes are distributed in two intervals, the first interval LAT1 is located in TR_L_ and U_L_, and the second interval LAT2 is located in the region where IR_L_ connects with U_L_ 37. A fragment of IR_L_ was expanded to reveal the location of LAT1 gene, overlapping ICP0 and ICP34.5, 8.3 kb original LAT (unstable), 2 kb and 1.5 kb LAT intron (stable). The direction of transcription is indicated by the black arrowhead. TATA indicates the location (in the genomic DNA) of the LAT1 promoter TATA box. The start of LAT1 transcription is indicated by +1, corresponding to nt 7569 of the genome. CRE-1 and CRE-2 denote the locations of the binding sites of the cAMP response elements 1 and 2 identified by Leib et al and Kenny et al 38, 39. LAT2 locus overlaps with the ICP0 and ICP34.5. The 8.3 kb primary LAT2 is spliced to an unusually stable 2.0 kb intron and a 6.3 kb short lived unconfirmed mRNA. Alternatively the 8.3 kb transcript can be spliced as Twintron introns to a 0.5 kb unstable intron and a 7.8 kb RNA (1.5 kb plus 6.3 kb) 40, 41. **B.** HSV-2 genome contains the LAT locus similar to HSV-1 in Figure 1A 37. LAT2 transcripts: 9.0 kb primary LAT (unstable), 2.2 kb major LAT (stable). The start of LAT2 transcription is indicated by +1, corresponding to nt 119507 of the genome.

As shown in Figure 1, genes encoding LATs have two intervals, called fragment 1 (LAT1) and fragment 2 (LAT2). LATs are spliced into non-major LAT (minor LAT or primary LAT) and major LAT. HSV-1 LATs are the transcription family of a group of RNAs, consisting mainly of 8.3 kb of low-level original transcripts and 3 introns. The 2 kb intron LAT generally presents at high levels during lytic and latent infection. The 1.5 kb intron LAT is detected during latency. The 0.5 kb LAT was not detected.^30^ The 6.3kb exon, which were not easily detected by Northern blot analysis 31, 32. Translation of a protein from the LAT exon mRNA has yet to be convincingly proved 33, nevertheless, it is obvious that exon 1 region of LAT is a vital part to prevent apoptosis 28, 34. LAT1 has two overlapping introns (2.0kb/1.5kb) produced by LAT transcripts, named to as double introns. Likewise, HSV-2 LAT gene was transcribed to generate about a 9.0 kb of non-primary LAT, and a stable 2.2 kb of main LAT. However, Transcripts of HSV-2 LAT1 are rarely reported. Both HSV-1 and HSV-2 have LAT promoter 1 (LAP1) and LAT promoter 2 (LAP2) 35. LAP1 is one of the most critical promoters for the efficient activation of LAT1s expression, which has been demonstrated in the incubation period of HSV infection. The same LAP2 has also been shown to have promoter activity, and the sequences in this region have enhanced HSV-1 LAP1 activity during a latent infection 36.

HSV latency in neurons is a major issue in the treatment of HSV infection and engineering of effective and safe oHSVs. Here we sequenced the LAT encoding DNA and LAT transcription regulatory region of our recently isolated new strain HSV-1-LXMW, and identified HSV LAT TRSs that bind to 9 transcription factors. Our results further illustrated HSV LAT biology and provided new options for better HSV treatment and oHSVs in the future.

## Materials and Methods

### LAT genomic DNA sequencing of our new strain HSV-1-LXMW

Our laboratory isolated a new HSV strain named HSV-1-LXMW from a male patient with oral herpes in Beijing, China 13. LAT DNA sequencing was carried out by Beijing institute of genomics and these sequences were analyzed using Burrows-Wheeler Aligner (BWA) software as described earlier 13. The genome sequences of the other 5 strains of HSVs were obtained from the NCBI reference database and summarized in table 1. Interestingly, we found that HSV-1 Strain Macl and HSV-2 strain H1226 did not have LAT1.

### Phylogenetic analysis of HSV LATs

The online MEGA7 software was used for phylogenetic analysis of TRRs of the LAT genes in 6 HSV strains. The evolution history of LAT is explored on the basis of time model by means of self-extracting uniform tree and maximum likelihood method. In 50% of boot replicates, the branch corresponding to the replicated partition forms a fold. Then, the original homologous tree of LAT can be obtained by combining the neighborhood join algorithm and BioNJ algorithm to estimate the distance matrix. Finally, the suitable topology of logarithmic likelihood value is selected.

### Genomic DNA sequencing of the transcription regulatory region (TRR) of our new strain HSV-1-LXMW

To determine the transcriptional regulation sequence of LAT gene, we extended by 2kb from the upstream of LAT gene as its TRR. For instance, taking the standard strain HSV-1-17 as an example, LAT1 is a reverse gene whose gene position is 1-7569bp, and 7569 is set as the transcription initiation site (represented by +1). TRR1 was chosen from 7569 to 9569 bp. LAT2 is a forward gene, whose position is 118777-127151bp. TRR2 was chosen from 116777 to 118777bp. Similarly, the TRRs of other HSV LAT genes were chosen (Table 2). LAT TRR genomic DNA sequencing of our original strain HSV-1-LXMW was performed as described earlier and above 13.

### Prediction of LAT transcription regulatory sequences (TRSs) and factors (TFs)

To predict LAT TRSs, TFs, we used the online program Matching. Match (http://gene-regulation.com/pub/programs.html) is designed to predict DNA transcription factor binding sites (TFBS) using a location-weight matrix library from TRANSFAC Public 6.0. The prediction was performed when all parameters were set to the strict default condition.

### Alignment of LAT TRRs of HSV-1 and HSV-2 strains

To know if there are conservative sequences among the LAT TRRs, we used the software ApE (http://www.bio-soft.net/plasmid/ApE.htm) to compare the 10 TRRs of LATs from 6 HSV strains at a setting of > 5 base pairs. The alignment parameters were set at default except blocks 10, mismatch penalty 0, gap penalty 0, gap ext penalty 0.

## Results

### LAT DNA sequences of our new strain HSV-1-LXMW and other HSV strains

The sequences of HSV-1-LXMW LAT were determined as follows: LAT1 from 1 to 7589bp, LAT2 from 118783 to 127151bp (see supplementary results 1, 2). Blast analysis showed that our HSV-1-LXMW LAT and HSV-1-17 LAT strains are highly similar, but our HSV-1-LXMW sequences are very different that of HSV-2 strains. Therefore, our data validated that the new strain of HSV-1-LXMW belongs to HSV-1 (Table 1).

### LAT phylogenetic analysis showed HSV-1-LXMW is close to strain HSV-1-17

**Figure 2:**
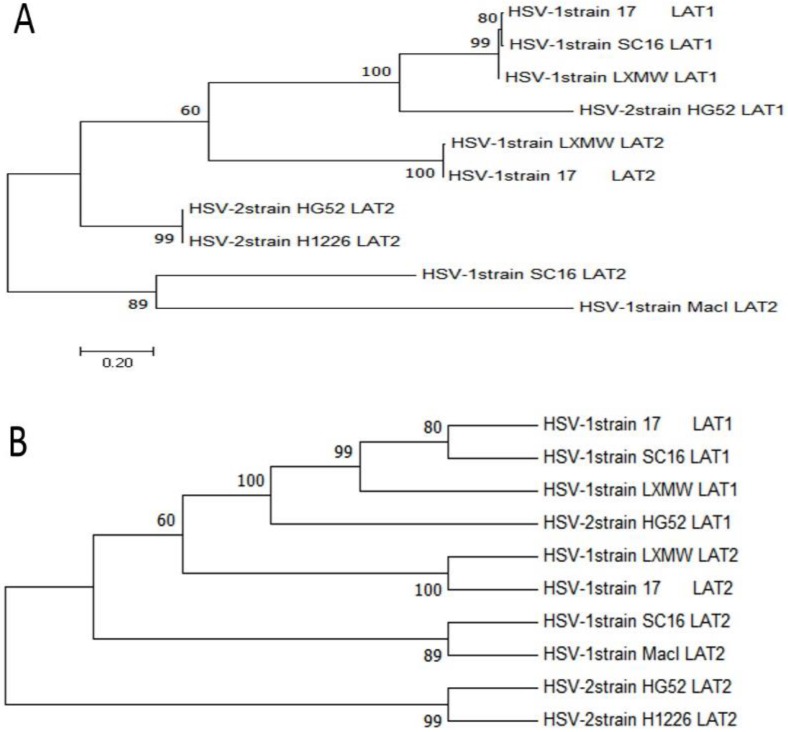
** Phylogenetic analysis of HSV-1-LXMW together with 5 other HSV strains. A.** Phylogenetic trees take the number of substituents at each site as the length of their branches and are plotted to a certain scale. **B.** The taxon was presented using the bootup consistent tree method, and the evolutionary history of the evolutionary tree was analyzed using MEGA7.

To study the evolutionary relationships of our new strain HSV-1-LXMW with other HSV-1 and HSV-2 strains, phylogenetic analysis was performed using MEGA7 software (http://www.liangchan.net/ liangchan/9113.html).The sequences of LAT1 and LAT2 of HSV-1-LXMW and 5 strains were analyzed. They include HSV-1-LXMW LAT1 and LAT2, HSV-1-17 LAT1 and LAT2, HSV-1-SC16 LAT1 and LAT2, HSV-1-Macl LAT2, HSV-2-HG52 LAT1 and LAT2, HSV-2-H1226 LAT2. Both the phylogenetic tree data (Figure 2) and neighbor network data showed the presence of four groups of clustering structures. Our new strain HSV-1-LXMW isolated in Beijing, China is close to the strain HSV-1-17 from MRC University, Glasgow, UK and far from the strain HSV-2-HG52 in University of Glasgow, UK and the strain HSV-2-H1226 in Pennslyvania State University, USA. The data showed a mean distance of approximately 52.7% among the strains tested collectively.

### The sequences of LAT transcription regulatory regions (TRRs) of our new strain HSV-1-LXMW and other strains

The sequences of LAT TRRs of new HSV-1- LXMW strain are 7589-9589 bp for TRR1, and 116797- 118797 bp for TRR2 (See Table 2, and supplementary results 3 and 4). The sequences of the LAT TRRs of the other 5 HSV strains studied in this article are listed in Table 2. It was found that the TRRs of LAT1 overlapped with part or all of the UL1 gene (envelope glycoprotein L), while the TRRs of LAT2 overlapped with part or all of the UL56 gene. Interestingly, the HSV-1-macl LAT2 transcriptional regulatory region overlaps with part of the RS1 gene (ICP4) as shown in Table 2.

### Potential LAT TRSs and TFs were identified

Discovery of TRSs and TFs of LAT is vital to understand the transcriptional regulation of LAT expression, HSV latency and oHSV engineering. Using the online program Matching, we found 9 potential TFs and their corresponding transcription regulatory sequences for the 6 strains of HSV LATs (Table 3). The nine transcription factors are Hairy, HFH-3, V-Myb, COMP1, E2F, TCF11/MafG, C-REL, Kr, AP-1.

### The LAT TRSs and TFs are conserved or unique

**Figure 3:**
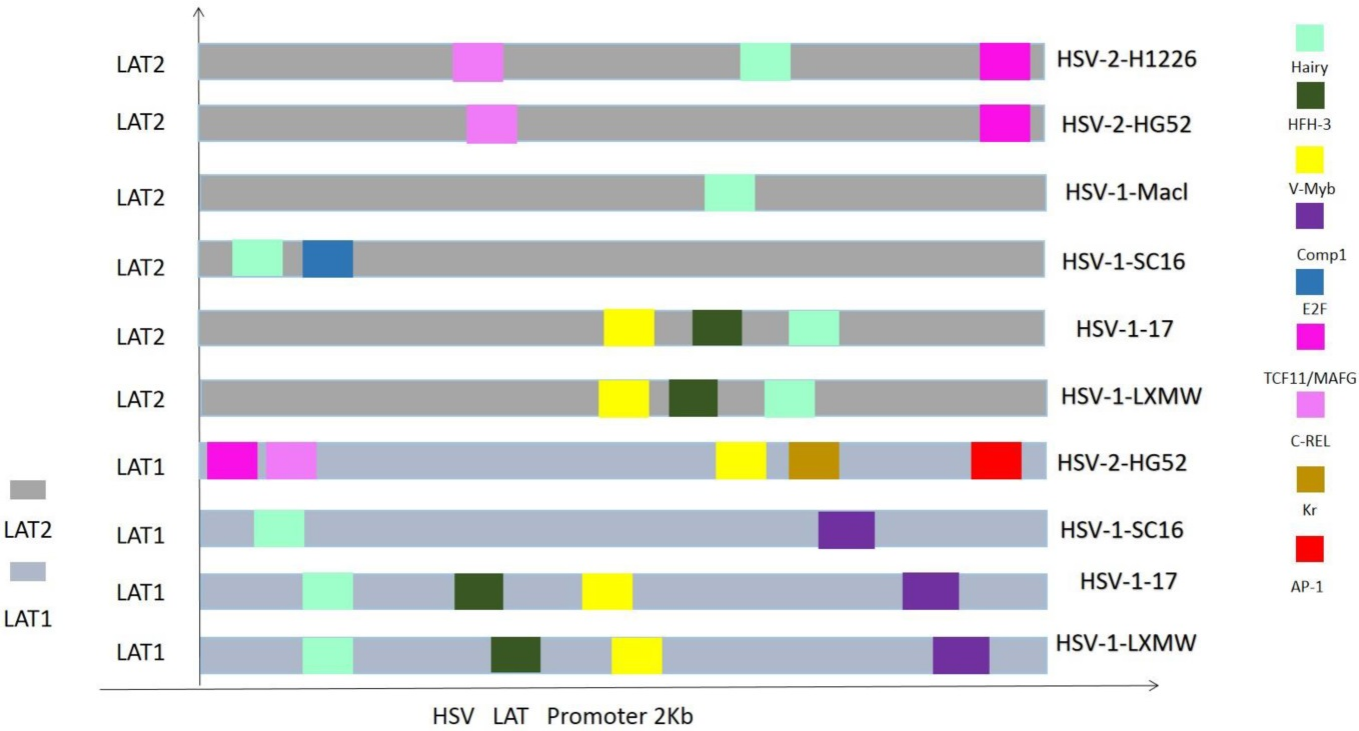
The LAT TRRs and TFs in HSVs.

**Figure 4:**
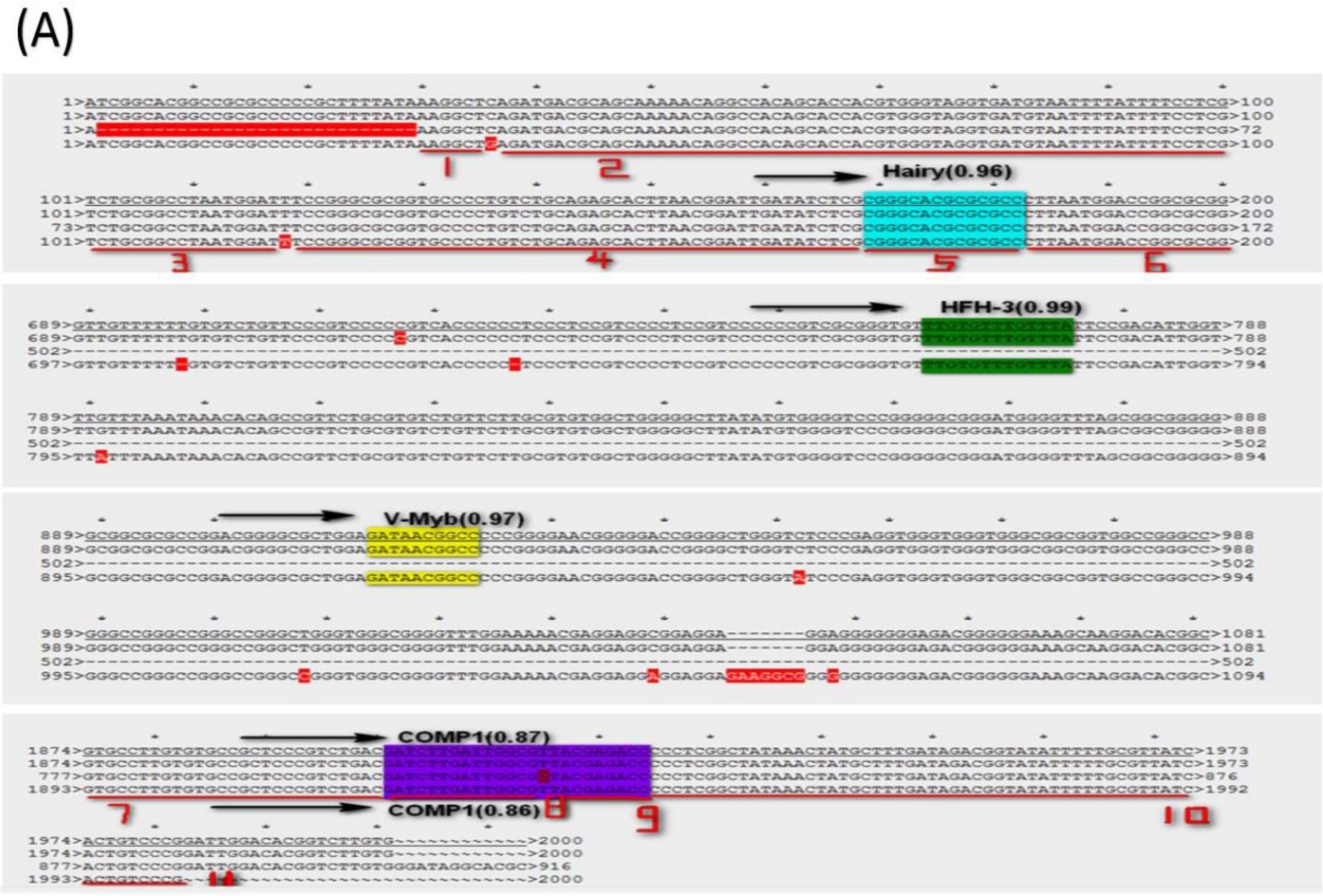

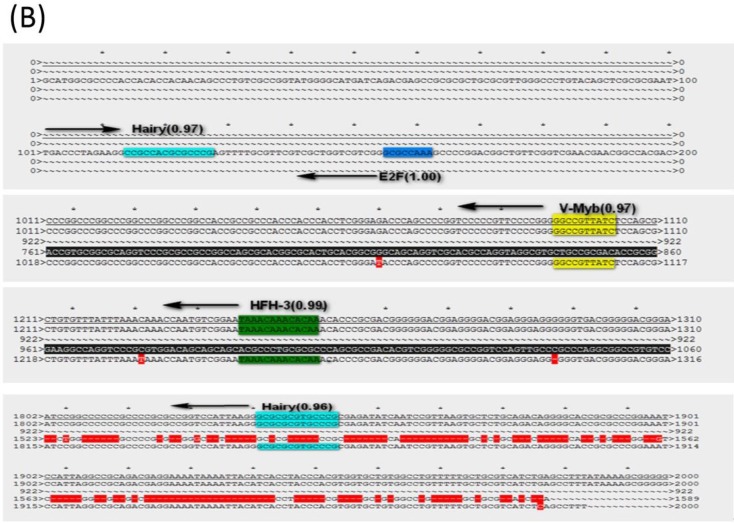

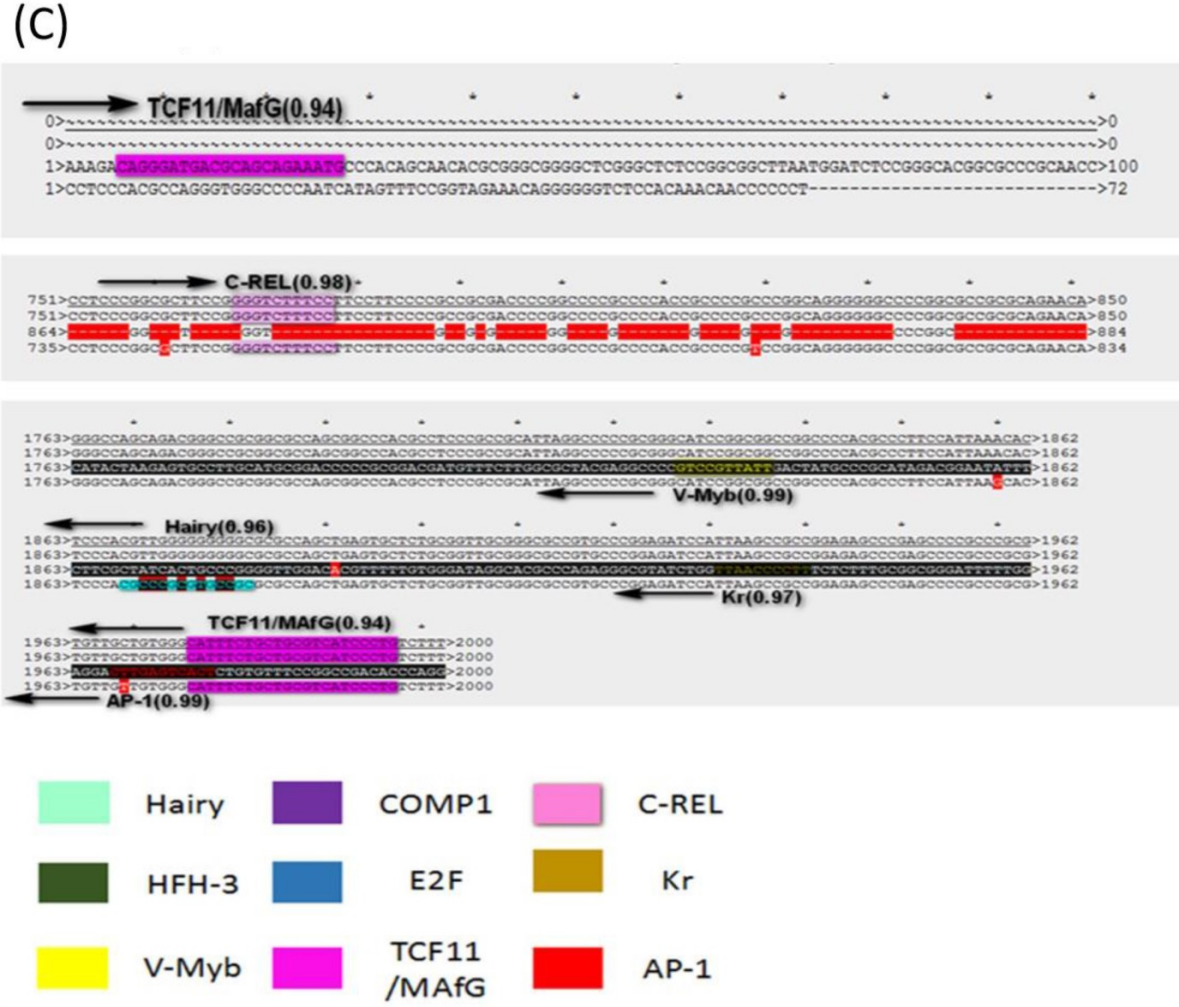
** The LAT transcription regulatory sequences and factors in HSVs. (A)** HSV-1-LXMW LAT1 TRR (7589-9589, 2000bp) alignment to those of HSV-1-LXMW, HSV-1-17 and HSV-1-SC16. When aligned to HSV-1-LXMW LAT1 TRR, HSV-1-17 and HSV-1-SC16 have 1963 and 887 matched base pairs, 14 and 5 mismatched base pairs, and 48 and 1133 gaps, respectively. There were 11 conserved regions starting from the 30 bp, 37 bp, 101 bp, 119 bp, 169bp, 183 bp, 1893 bp, 1922bp, 1937bp, 1946 bp and 1993bp of the HSV-1-LXMW TRR, and four conserved TRSs binding to the four TFs of Hairy, HFH-3, V-Myb and comp1, respectively. **(B)** HSV-1-LXMW LAT1 TRR (116797-118797, 2000bp) alignment to those of HSV-1-LXMW, HSV-1-17, HSV-1-SC16 and Macl. When aligned to HSV-1-LXMW LAT1 TRR, HSV-1-17, HSV-1-SC16 and HSV-1-Macl have 1969, 424 and 96 matched base pairs, 14, 2 and 3 mismatched base pairs, and 36, 2071 and 162 gaps, respectively. There were four conserved TRSs binding to the four TFs of Hairy, E2F, v-myb, and HFH-3, respectively. **(C)** HSV-2-HG52 LAT2 TRR (117507-119507, 2000bp) alignment to those of HSV-1-HG52 LAT2, HSV-1-HG52 LAT1 and HSV-2-H1126 LAT2. When aligned to HSV-1-HG52 LAT2 TRR, HSV-1-HG52 LAT1 and HSV-2-H1126 LAT2 have 331 and 1881 matched base pairs, 19 and 13 mismatched base pairs, and 1003 and 214 gaps, respectively. There were six TRSs binding to the six TFs of TCF11/MAfG, C-REL, V-Myb, Hairy, Kr, AP-1, respectively.

To know if the predicted LAT TRSs and TFs are conserved among HSVs and thus likely to be biologically functional, we did sequence alignment of the TRRs of LAT1 and LAT2 of HSV-1 and HSV-2 genomes (Figure 3 and 4). The identified transcription factors and their relative positions in the TRRs of LAT1 and LAT2 of HSV-1 and HSV-2 genomes are shown in Figure 3. Among all transcription factors, Hairy is shared for all LATs of HSV1 and HSV-2 except the strain HSV-2-HG52. Furthermore, HFH-3, V-Myb, Comp1, Kr and E2F are found only in HSV-1 strains; while C-REL, TCF11/MafG and AP-1 are found only in HSV-2 strains. In addition, Comp1, Kr and AP-1 are found only for LAT1; while E2F is found only for LAT2.

Because LAT TRRs of HSV-1 and HSV-2 are very diverse, and the two segments of LAT genes (LAT1 and LAT2) have different directions, we compared LAT TRRs in three groups (group a, b and c), using ApE Program. Group A: All HSV-1 LAT1; Group B: All HSV-1 LAT2; Group C: HSV-2 LAT1, LAT2. We found that the TRRs of our new strain HSV-1-LXMW are highly similar to the strain HSV-1-17. Our data also showed that the LAT TRRs are conserved within each group, and the TRSs identified above were mostly overlapped with the conserved sequences with certain exceptions (Figure 4).

The conservation of the identified TRSs and TFs suggests that these may have biological functions; meanwhile HSV-1 or 2 specific and LAT-1 or 2 specific sequences and factors may be related to their specific biological function. However, to validate these, further functional studies are needed.

### The transcription factors v-myb and AP-1 family JunD are functional in regulating HSV gene transcription, including LATs transcription for HSV infection and latency

To validate if the identified TRSs and TFs are actually functional in HSV infected cells, we searched the literature that reported each of the 9 TFs in HSV infected cells, and found that the transcription factors v-myb and AP-1 are functional in regulating HSV gene transcription, including LATs transcription for HSV infection and latency (Table 4), and no report of the other 7 TFs in HSV infected cells.

In HSV-1, v-myb has been reported to activateγ34.5 42, 43 and inhibit ICP6 44 gene transcription. Interestingly, the gene encoding γ34.5 overlaps with the genes for LATs. Studies have also shown that v-myb activates the transcription of thymidine kinase (TK) gene 45. TK is required for HSV DNA replication, lytic infection and reactivation from latent infection 46. The absence of TK limits virus replication in non-dividing cells, such as ganglia neurons 47.

According to the literature, AP-1 family JunD can bind and activate the promoter of LAT1 48. HSV-1 LAT transcription regulatory cyclic-AMP (cAMP) response element (CRE)-like sequences, CRE-1 and CRE-2, were shown to regulate latency reactivation and support basal LAT expression in neuronal cells, respectively. Suggesting that the AP-1 family of transcription factors function in regulating CRE-dependent LAT1 transcription activation. The reaction of sensory ganglia to HSV infection is consistent with the neurobiological responses observed during nerve regeneration of peripheral processes following injury, including the alterations of neuropeptide production 49, neurite sprouting 50, and upregulation of AP-1 factors 51. It is possible that these regenerative responses are linked to signaling pathways that regulate LAT transcription activity. In addition, HSV-2 can induce AP-1 transcriptional activation through the HSV replication affecting TLR4-MyD88/TRIF pathway in human genital epithelial cell 52.

However, these predicted transcription factors above have not been reported to regulate LAT gene transcription, which requires further study in the future.

## Discussion

Recent studies have found that high expression of HSV LAT in both the human trigeminal nerve and the eye is associated with latent infection of HSV 53. Deletion of the LAT gene can significantly reduce the latent infection rate and the activation of the virus 54. Studies have shown that HSV-1 recombinants with deletions in the LAT promoter and portions of the 5' exon coding region lead an increase in apoptotic neurons during the acute infection 55, resulting in a 2-3 fold decrease in total HSV-1 DNA detected in the ganglia during latency in the rabbit eye 56. The deletion of both LAP2 and LAP1 eliminated the ability to detect LAT in acute and latent infections. The deletion of LAP2 alone diminished levels of LAT expression in both acute and latent infections 57. Thus, sequences important for wild-type-level LAT transcription during productive infections and during latency appear to reside within LAP2. To make LAP2 deletions, most of the sequences between the 5' end of the primary LAT and the 5' end of the LAT intron (positions 4081 to 4382) were deleted 58.

Interestingly, a consistent effect of the LAP2 deletion on LAT expression was observed in every experiment, including those performed during the productive infection of Vero and human neuroblastoma cells and with the latently infected ganglia 57. More recently, studies have shown that HF10 (an oHSV with natural deletions: the UL56/IRL junction has been deleted from 116.515 bp to 120.346 bp, leading to the lack of expression of UL56 and LATs 59. It is worth noting that the deleted sequence in HF10 overlaps with the LAT2 regulatory sequence we studied (116.797 bp to 118.797 bp) in our strain HSV-1-LXMW. This suggests that the HSV LAT sequences we studied is of significance for HSV LAT transcription and oHSVs. Actually clinical studies on HF10 have been completed in breast, head and neck, and pancreatic cancers in Japan 59, 60. Meanwhile, in the United States, HF10 has completed phase I clinical trials for refractory superficial cancers and melanoma 59. Importantly, HF10 has been shown to have a high safety profile and low adverse reactions in all patients treated 59. HF10 viral replication and cytotoxicity has also been studied in human and mouse melanoma cell lines 59. Our newly discovered transcription factors and their corresponding transcription regulatory sequences may be used to effectively regulate the expression of LAT, to control HSV latency for developing safer oHSVs in the future. In this article, for the first time, we did systematic comparative sequence analysis of LATs and their TRRs from 6 different HSV-1 and HSV-2 strains. Our important findings include the following.

Our recently isolated new strain HSV-1-LXMW was found to be closely related to HSV-1-17 from MRC University, Glasgow, UK and SC16 from the Spain, which is different from the ICP27-based results 13, and different from other methods using PCR 61, FISH 62, ISH 63, Northern blot analysis 64. The chimeric virus HSV-1-17/LAT2, which is an HSV-1 virus engineered to express HSV-2 LAT (complementary to HSV-2 333/LAT1) preferentially establishes latent infections in KH10-positive neurons, as does wild-type HSV-2 65. Therefore, it is likely that our newly isolated HSV-1-LXMW strains can be used to establish a latent model and build a platform for further study on the virus latency mechanism since it's highly homologous to HSV-1-17.

We identified novel conservative HSV LAT transcription regulatory sequences and 9 potential LAT transcription factors of Hairy, HFH-3, v-Myb, Comp1, E2F, TCF11/MAFG, C-REL, Kr and AP-1. Previous studies analyzed fewer HSV strains and shorter DNA sequences (2000 vs. 900 nt) 57 66 67 and reported only two LAT transcription factors v-myb 42-45 and AP-1^52^, ^68^. For the first time, we discovered seven novel transcription factors and their corresponding transcription regulatory sequences of HSV LATs. Our findings may have significant implication in our understanding of HSV latency and engineering of better oncolytic HSVs. Among them v-Myb and AP-1 were reported to be functional in HSV infected cells, while the other 7 were reported here for the first time. Their functions in HSV1/2 tissue tropism and latency need to be further explored. We summarized the HSV-1/2 tissue tropism and the TFs expression in a variety of tissues (Table 5). The table shows in detail that most of our 7 transcription factors are moderately expressed in HSV-1 trigeminal ganglion and moderately or highly expressed in HSV-2 reproductive system. We propose that the variable expression of the transcription factors may contribute to the LAT transcription in different tissues, which may be related to the HSV latency in the proper cells.

Hairy is a developmental suppressor gene and a crucial part of normal body development and nervous system development 69. HFH-3 is a cell-type-specific transcription factor 70. v-Myb with DNA-binding, activation and repression domains, interacts with the CAAT-enhancer binding protein (C/EBP) family, and regulates proliferation and differentiation of hematopoietic cells 71. The cellular transcription factor E2F, initially identified as a target for transactivation by adenovirus ElA 72, 73, is found in all kind of cell types. TCF11 and the Maf proteins -F, -G and -K/p18 are widely expressed, and interact with each other in numerous cell types 74. C-Rel is a protooncogene product that may be involved in the transcriptional regulation of the family of genes that contain this ISRE 75. AP-1 is a big family that includes Jun and Fos 76. The results indicate that at least one neuronal AP-1 family member, JunD, is involved in a major fraction of the LAP1 CRE binding activity 77. JunD has been observed to be induced by treatment of myeloid cell lines with a cAMP analog 48. AP-1 activates caspase 8 transcription, which mediates palmitic acid induced apoptosis of human cardiac myocytes, leading to diabetic cardiomyopathy 78. Because partial core promoter region was not included in our analysis, transcription factor AP-1 binding site was not detected in HSV-1 strains. However AP-1 family JunD was reported to bind to the CRE elements in the core promoter region.

**Figure 5:**
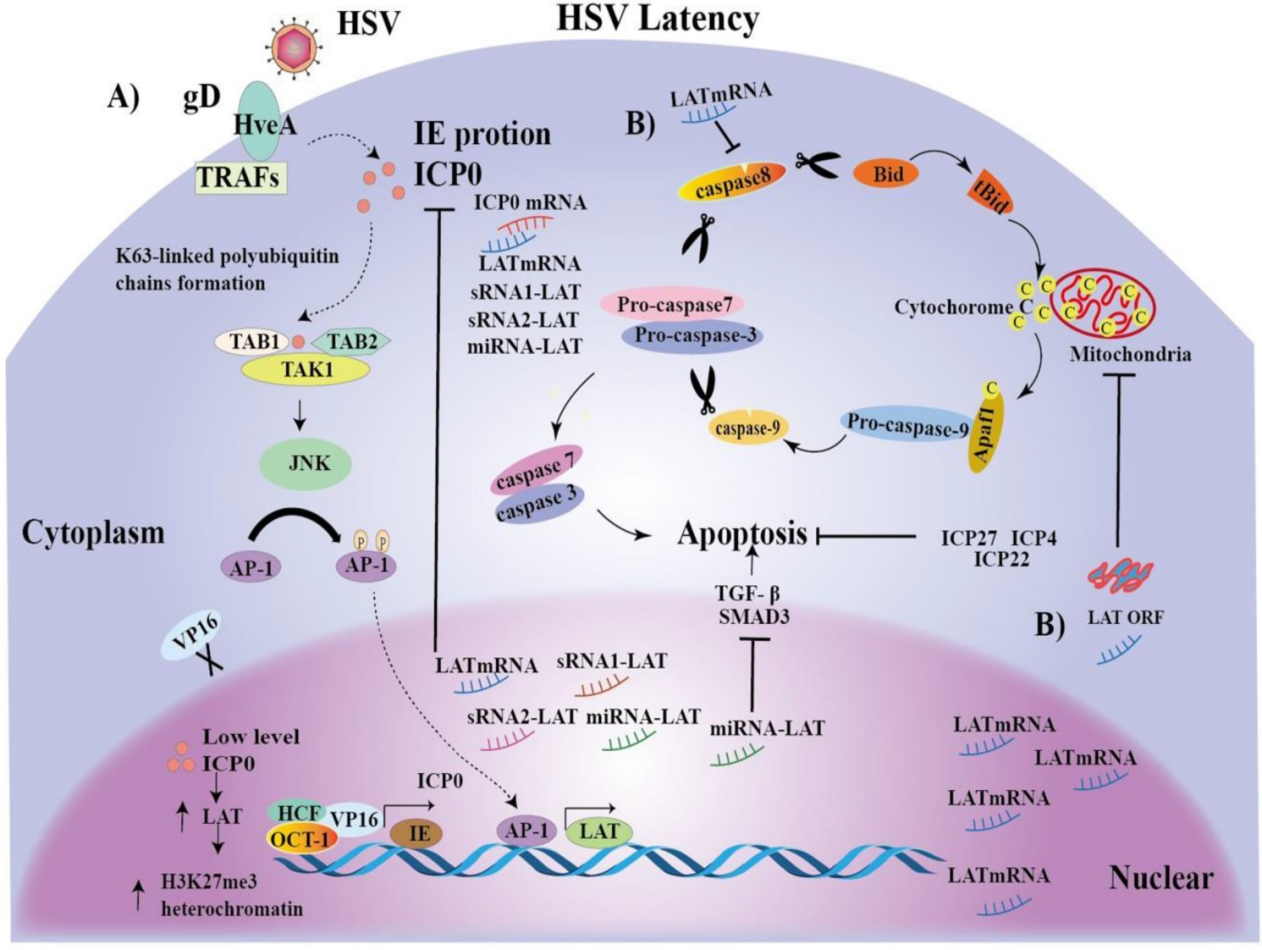
** Mechanisms of the initiation and maintenance of HSV latent infection. A)** The HSV envelope glycoprotein gD binds to the TNFR member HSV entry mediator A (HveA), whose cytoplasmic region binds to TRAFs 79. Upon HSV entry, multiprotein complex VP16, Oct-1 and HCF initiate IE gene transcription, including ICP0, promoting lytic infection 80. In turn, ICP0, together with Ubc13ev1A, catalyzes the K63-linked polyubiquitin chains, and recognizes and activates TAK1 80. Activated TAK1 then phosphorylates MKK6, leading to the activation of JNK kinase 80. Activated TAK1 then phosphorylates MKK6, leading to the activation of JNK kinase 80. Ap-1 transcription factors are phosphorylated by JNK and traffic into the nucleus to bind to the LAT transcription regulatory sequences, and, promote LAT transcription. LAT increases accumulation of H3K27me3 heterochromatin, leading to gene silencing and latency initiation 17. Then, a large number of LAT transcripts, such as mRNA, miRNA-LAT and sRNA1-LAT or sRNA2-LAT, inhibit ICP0 gene expression by partial base complementation to maintain latency. ICP0 can also be reduced by VP16 loss. **B)** On the other hand, LAT expression inhibits caspase 8 and LAT ORF inhibits caspase 9, promoting cell survival 81. Interestingly, a miRNA-LAT generated from the exon 1 region of LATs was found to exert an anti-apoptotic effect through targeting transforming growth factor beta (TGF-β) and SMAD3 expression. Finally, the regulatory proteins ICP4, ICP22 and ICP27 may indirectly inhibit apoptosis by promoting the production of anti-apoptotic viral products.

In a word, AP-1 may promote LAT transcriptional expression and latency. The mechanisms of LAT-mediated latent infection have been proposed as many hypotheses, but it is still unclear. To better understand the significance of our identification of the novel LAT TRSs and TFs, we integrated reported hypothesis and our novel findings, and proposed possible mechanisms for the initiation and maintenance of HSV latent infection (Figure 5).

## Supplementary Material

Supplementary results.

## Figures and Tables

**Table 1:** HSV LAT genomic DNA sequencing.

HSV strain	Gene Bank ID	Tax-ID	Sub-Date	LAT DNA sequence	Start site of transcription (+1)	University, Country
**HSV-1** **strain LXMW**				LAT1 (1—7589) LAT2(118783-127138)	LAT1 (7589→1) LAT2(118812-127138)	Yangtze University, Jingzhou, China
**HSV-1** **strain 17**	JN555585.1	10299	2011-08-02	LAT1 (1--7569) LAT2(118777-127151)	LAT1 (7569→1) LAT2(118805→127151)	MRC University, Glasgow, UK
**HSV-1** **isolate SC16**	KX946970.1	10309	2016-10-30	LAT1(1-8251) LAT2 (118713-126490)	LAT1(8223→1) LAT2 (119234-126490)	SeveroOchoa, Spain
**HSV-1** **Strain Macl**	KM222720.1	10298	2014-7-21	LAT2 (118188-126929)	LAT2(118216-126929)	Pennslyvania State University, USA
**HSV-2** **strain HG52**	JN561323.2	10315	2011-08-05	LAT1 (1-7814) LAT2 (119507-127991)	LAT1 (7767→1) LAT2 (119554→127991)	University of Glasgow, UK
**HSV-2** **strain H1226**	KY922720.1	10310	2017-4-06	LAT2 (119356-128342)	LAT2 (119403→128342)	Pennslyvania State University, USA

**Table 2:** The sequences of the LAT TRRs of our new strain HSV-1-LXMW and other strains.

HSV Strain	Gene (bp)	The direction of the promoter	transcriptional regulation sequence(2000bp)	The closest gene to LAT	Overlap sequence(bp)
**HSV-1--LXMW**	LAT1	→	7589-9589	none	none
	LAT2	←	118797-116797	none	none
**HSV-1-17**	LAT1	→	7569-9569	UL1 (9338-10949)	9338-10949 (Overlap 1611)
	LAT2	←	118777-116777	UL56 ( 116197-116926)	116926-116777 (Overlap 729)
**HSV-1-SC16**	LAT1	→	8251-10251	UL1 (9989-11600)	9989-10251 (Overlap 262)
	LAT2	←	118713-116713	UL56 (116630-117359)	116713-117359 (Overlap 646)
**HSV-1-Macl**	LAT2	→	126929-128929	RS1 (126951-131250)	126951-128929 (Overlap 1978)
**HSV-2-HG52**	LAT1	→	7814-9814	UL1 (9463-11060)	9463-9814 (Overlap 351)
	LAT2	←	119507-117507	UL56 (117079-117817)	117507-117817 (Overlap 310)
**HSV-2-H1226**	LAT2	←	119356-117356	UL56 (117000-117738)	117738-117356 (Overlap 382)

**Table 3:** LAT transcription regulatory sequences and factors in HSV.

HSV strain	Matrix identifier	Position on the genome	Core match	Matrix match	Sequence	Factor name	
**HSV-1** **strain LXMW**	I$HAIRY_01	7759	1.000	0.967	cgggCACGCgcgcc	Hairy	LAT1
	V$HFH3_01	8352	1.000	0.994	ttgTGTTTgttta	HFH-3	
	V$VMYB_01	8503	1.000	0.972	gatAACGGcc	v-Myb	
	V$COMP1_01	9490	1.000	0.874	gatcttGATTGgcgttacgagacc	COMP1	
	V$VMYB_01	117689	1.000	0.972	ggCCGTTatc	v-Myb	LAT2
	V$HFH3_01	117541	1.000	0.994	taaacAAACAcaa	HFH-3	
	I$HAIRY_01	116948	1.00	0.967	ggcgcGCGTGcccg	Hairy	
**HSV-1** **strain 17**	I$HAIRY_01	7738	1.000	0.967	cgggCACGCgcgcc	Hairy	LAT1
	V$HFH3_01	8338	1.000	0.994	ttgTGTTTgttta	HFH-3	
	V$VMYB_01	8489	1.000	0.972	gatAACGGcc	v-Myb	
	V$COMP1_01	9489	1.000	0.874	gatcttGATTGgcgttacgagacc	COMP1	
	V$VMYB_01	117676	1.000	0.972	ggCCGTTatc	v-Myb	LAT2
	V$HFH3_01	117528	1.000	0.994	taaacAAACAcaa	HFH-3	
	I$HAIRY_01	116929	1.000	0.967	ggcgcGCGTGcccg	Hairy	
**HSV-1** **strain SC16**	I$HAIRY_01	8392	1.000	0.967	cgggCACGCgcgcc	Hairy	LAT1
	V$COMP1_01	10140	1.000	0.866	gatcttGATTGgcgctacgagacc	COMP1	
	I$HAIRY_01	118827	1.000	0.973	ccgcCACGCgcccg	Hairy	LAT2
	V$E2F_02	118558	1.000	1.000	gCGCCAaa	E2F	
**HSV-1** **strain Macl**	I$HAIRY_01	119524	1.000	0.972	gccgCACGCggcct	Hairy	LAT2
**HSV-2** **strain HG52**	V$TCF11MAFG_01	7820	1.000	0.948	cagggATGACgcagcagaaatg	TCF11/MafG	LAT1
	V$CREL_01	6588	1.000	0.982	GGAAAgaccc	c-Rel	
	V$VMYB_01	5988	1.000	0.990	gtCCGTTatt	v-Myb	
	I$KR_01	5884	1.000	0.975	ttAACCCctt	Kr	
	V$AP1_Q4	5847	1.000	0.990	cttgAGTCAct	AP-1	
	V$CREL_01	120274	1.000	0.982	gggtcTTTCC	c-Rel	LAT2
	V$TCF11MAFG_01	117532	1.000	0.948	catttctgctgcGTCATccctg	TCF11/MafG	
**HSV-2** **strain H1226**	V$CREL_01	120107	1.000	0.982	gggtcTTTCC	c-Rel	LAT2
	I$HAIRY_01	117488	1.000	0.962	cgcccGCGTGccgc	Hairy	
	V$TCF11MAFG_01	117381	1.000	0.948	catttctgctgcGTCATccctg	TCF11/MafG	

**Table 4:** The transcription factors v-myb and AP-1 family JunD are functional in regulating HSV gene transcription

Tissue type	HSV	V-Myb	AP-1	TF function	Ref
Brain: Human glioblastoma cells	HSV-1-F	B-Myb	---	activate/γ34.5	42
Urinary: Human PDAC-derived Capan-2 cells, BxPC-3, pancreatic	HSV-1-F	Myb	---	Inhibit/ICP6	44
Colon: Human HT29 carcinoma cells,	HSV-1-F	Myb	---	activate/γ34.5	43
The bone marrow: myelomonocytic hematopoietic cells, NIH 3T3 cells.	HSV-1- McKrae	v-myb	---	activate/TK	45
Neuronal: neuroblastoma C1300 Cell	HSV-1- 17	---	AP-1 family JunD	activate/LAT1	48
Genital epithelial cells HEC-1-A	HSV-2	---	not reported	activate/AP-1	52

**Table 5:** The HSV-1/2 tissue tropism and the TF expression from all sorts of tissues.

System	Cell/ tissue	HSV1	HSV2	Hairy	HFH-3	v-Myb	E2F	TCF11/MAfG	C-REL	AP-1
**Blood system**	CD34 + stem cell	+	_	N	M	M	H	L	H	M
	721 B lymphoblasts	+	_	N	L	M	M	M	H	M
	CD19 + B cell	+	_	H	L	M	H	L	H	H
	Leukemia lymphoblastic	+	_	M	L	H	M	L	M	L
	Bonemarrow	+	_	M	M	M	L	L	H	L
	Pituitariy	+	_	H	M	H	L	M	H	L
**Head**	Prefrontal Cortex	+	_	H	L	M	M	M	H	H
	Pineal	+	_	H	L	H	H	H	H	M
	Tongue	+	_	M	M	H	L	M	H	L
	Tonsil	+	_	M	L	M	M	L	H	L
	Retina	+	_	H	M	H	M	H	H	M
	Trigeminal ganglion	+	_	L	L	M	L	M	M	M
	Cerebellum	+	_	M	M	M	L	L	H	L
**viscera**	Heart	+	_	H	M	M	L	M	H	M
	Lung	+	_	H	M	M	L	M	H	H
	Liver	+	_	H	M	M	M	L	H	M
	Kidney	+	_	H	L	M	L	M	M	L
	Smooth Muscles	+	_	H	L	M	L	M	H	L
	Adipocyte	+	_	H	M	M	L	M	H	L
**Secretory system**	Adrenalgland	+	_	H	L	M	L	M	M	M
	Pancreaticlstet	+	_	H	M	M	M	L	H	M
**Genital system**	Placenta	+	+	H	L	H	M	M	H	L
	Fetalthyroid	+	+	H	M	M	L	L	H	L
	Uterus	+	+	M	L	M	M	M	M	M
	Testis	+	+	M	M	M	M	M	M	L

Hold increase to median fluorescence intensity on Affymetrix microarray chips from http://biogps.org: 0-2.5 (L), >2.5-<5 (M), >5 (H)

## Abbreviations

HSV: Herpes simplex viruses
oHSV: oncolytic herpes simplex virus
LATs: Latency-Associated Transcripts
TRRs: transcription regulatory regions
TRSs: Transcription Regulatory Sequences
TFs: Transcription Factors
TFBS: transcription factor binding sites
LAP1: LAT promoter 1
LAP2: LAT promoter 2
UL: Unique Long
US: Unique Short
TRL: Terminal Repeat Long
IRL: Internal Repeat Long
IRS: Internal Repeat Short
TRS: Terminal Repeat Short
IE: immediate-early
HSV-1-LXMW: strain LXMW of type 1 HSV
ICP: Infected Cell Polypeptide
UL1: envelope glycoprotein L
TK: thymidine kinase
HveA: HSV entry mediator A
JNK: c-Jun N-terminal kinase
AP-1: activator protein 1
TAK1: transforming growth factor-h-activated protein kinase 1
TRAFs: TNF receptor-associated factors
TGF-β: transforming growth factor beta
