# Supplementary material for: Transcriptional Regulation of Latency-Associated Transcripts (LATs) of Herpes Simplex Viruses
Source: J Cancer. 2020 Mar 5;11(11):3387–99. doi: 10.7150/jca.40186 (PMC7097949; doi:10.7150/jca.40186)
Supplement: Supplementary file 1 — Supplementary results. [file jcav11p3387s1.pdf]

supplementary results 1

New strain HSV-1-LXMW LAT1 From 1 to 7589bp.

AGCCCGGGCCCCCCGCGGGCGCGCGCGCGCAAAAAAGGCGGGCGGCG
GTCCGGGCGGCGTGCGCGCGCGCGGCGGGCGTGGGGGGCGGGGCCGCGG
GAGCGGGGGGAGGAGCGGGGGGAGGAGCGGGGGGAGGAGCGGGGGGAG
GAGCGGGGGGAGGAGCGGGGGGAGGAGCGGGGGGAGGAGCGGGGGGAG
GAGCGGGGGGAGGAGCGGGGGGAGGAGCGGGGGGAGGAGCGGGGGGAG
GAGCGGGGGGAGGAGCGGGGGGAGGAGCGGGGGGAGGAGCGGGGGGAG
GAGCGGGGGGAGGAGCGGGGGGAGGAGCGGCCAGACCCCGAAAACGGG
CCCCCCCCCAAAACACACCCCCCGGGGGTCGCGCGCGGCCCTTTAAAGCG
CGGCGGCGGGCAGCCCGGGCCCCCCGCGGCCGAGACTAGCGAGTTAGACA
GGCAAGCACTACTCGCCTCTGCACGCACATGCTTGCCTGTCAAACTCTACC
ACCCCGGCACGCTCTCTGTCTCCATGGCCCGCCGCCGCCGCCATCGCGGCC
CCCGCCGCCCCCGGCCGCCCGGGCCCACGGGCGCGGTCCCAACCGCACAG
TCCCAGGTAACCTCCACGCCCAACTCGGAACCCGTGGTCAGGAGCGCGCC
CGCGGCCGCCCCGCCGCCGCCCCCCGCCAGTGGGCCCCCGCCTTCTTGTTC
GCTGCTGCTGCGCCAGTGGCTCCACGTTCCCGAGTCCGCGTCCGACGACG
ACGACGACGACGACGACTGGCCGGACAGCCCCCCGCCCGAGCCGGCGCC
AGAGGCCCGGCCCACCGCCGCCGCCCCCCGCCCCCGGTCCCCACCGCCCG
GCGCGGGCCCGGGGGGCGGGGCTAACCCCTCCCACCCCCCCTCACGCCCC
TTCCGCCTTCCGCCGCGCCTCGCCCTCCGCCTGCGCGTCACCGCAGAGCAC
CTGGCGCGCCTGCGCCTGCGACGCGCGGGCGGGGAGGGGGCGCCGGAGC
CCCCCGCGACCCCCGCGACCCCCGCGACCCCCGCGACCCCCGCGACCCCC
ACGCGGGTGCGCTTCTCGCCCCACGTCCGGGTGCGCCACCTGGTGGTCTG
GGCCTCGGCCGCCCGCCTGGCGCGCCGCGGCTCGTGGGCCCGCGAGCGGG
CCGACCGGGCTCGGTTCCGGCGCCGGGTGGCGGAGGCCGAGGCGGTCATC
GGGCCGTGCCTGGGGCCCGAGGCCCGTGCCCGGGCCCGGGCCCTGGCCCG
CGGAGCCGGCCCGGCGAACTCGGTCTAACGTTACACCCGAGGCGGCCTGG
GTCTTCCGCGGAGCTCCCGGGAGCTCCGCACCAAGCCGCTCTCCGGAGAG
ACGATGGCAGGAGCCGCGCATATATACGCTTGGAGCCGGCCCGCCCCCGAG
GCGGGCCCGCCCTCGGAGGGCGGGACTGGCCAATCGGCGGCCGCCAGCGC
GGCGGGGCCCGGCCAACCAGCGTCCACCGAGTCGTTGGGGCCCGGCCCAC
TGGGCGGTAACTCCCGCCCAGTGGGCCGGGCCGCCCACTTCCCGGTATGGT
AATTAAAAACTTGCAGAGGCCTTGTTCCGCTTCCCGGTATGGTAATTAGAA
ACTCATTAATGGGCGGCCCCGGCCGCCCTTCCCGCTTCCGGCAATTCCCGC
GGCCCTTAATGGGCAACCCCGGTATTCCCCGCCTCCCGCGCCGCGCGTAAC
CACTCCCCTGGGGTTCCGGGTTATGTTAATTGCTTTTTTGGCGGAACACACG
GCCCCTCGCGCATTGGCCCGCGGGTCGCTCAATGAACCCGCATTGGTCCCC
TGGGGTTCCGGGTATGGTAATGAGTTTCTTCGGGAAGGCGGAAGCCCCG
GGGCACCGACGCAGGCCAAGCCCCTGTTGCGTCGGCGGGAGGGGCATGCT
AATGGGGTTCTTTGGGGGACACCGGGTTGGTCCCCCAAATCGGGGGCCGG
GCCGTGCATGCTAATGATATTCTTTGGGGGCGCCGGGTTGGTCCCCGGGGA
CGGGGCCGCCCCGCGGTGGGCCTGCCTCCCCTGGGACGCGCGGCCATTGG
GGGAATCGTCACTGCCGCCCCTTTGGGGAGGGGAAAGGCGTGGGGTATAA

GTTAGCCCTGGCCCGACGGTCTGGTCGCATTTGCACCTCGGCACTCGGAGC
GAGACGCAGCAGCCAGGCAGACTCGGGCCGCCCCCTCTCCGCATCACCAC
AGAAGCCCCGCCTACGTTGCGACCCCCAGGGACCCTCCGTCCGCGACCCT
CCAGCCGCATACGACCCCCATGGAGCCCCGCCCCGGAGCGAGTACCCGCC
GGCCTGAGGGCCGCCCCCAGCGCGAGGTGAGGGGCCGGGCGCCATGTCTG
GGGCGCCATATTGGGGGGCGCCATATTGGGGGGCGCCATGTTGGGGGACCC
CCGACCCTTACACTGGAACCGGCCGCCATGTTGGGGGACCCCCACTCATAC
ACGGGAGCCGGGCGCCATGTTGGGGCGCCATGTTAGGGGGCGTGGAACCC
CGTGACACTATATATACAGGGACCGGGGGCGCCATGTTAGGGGGCGCGGAA
CCCCCTGACCCTATATATACAGGGACCGGGGTCGCCCTGTTAGGGGTCGCCA
TGTGACCCCCTGACTTTATATATACAGACCCCCAACACATACACATGGCCCC
TTTGACTCAGACGCAGGGCCCGGGGTCGCCGTGGGACCCCCCCCCCCTG
ACTCATACACAGAGACACGCCCCCACAACAAACACACAGGGACCGGGGTC
GCCGTGTTAGGGGGCGTGGTCCCACTGACTCACACGCAGGGCCCCCTTAG
TCACACGCATCTAGGGGGGTGGGGAGGAGCCGCCCGCCATATTTGGGGGA
CGCCGTGGGACCCCCGACTCCGGTGCGTCTGGAGGGCGGGAGAAGAGGG
AAGAAGAGGGGTCGGGATCCAAAGGACGGACCCAGACCACCTTTGGTTGC
AGACCCCTTTCTCCCCCCTCTTCCGAGGCCAGCAGGGGGGCAGGACTTTGT
GAGGCGGGGGGGAGGGGGAACTCGTGGGCGCTGATTGACGCGGGAAATC
CCCCCCATTCTTACCCACCCCCCCCTTTTTTCCCCTTAGCCCGCCCCGGATGT
CTGGGTGTTTCCCTGCGACCGAGACCTGCCGGACAGCAGCGACTCGGAGG
CGGAGACCGAAGTGGGGGGGCGGGGGGACGCCGACCACCATGACGACGA
CTCCGCCTCCGAGGCGGACAGCACGGACACGGAACTGTTCGAGACGGGG
CTGCTGGGGCCGCAGGGCGTGGATGGGGGGGCGGTCTCGGGGGGGAGCC
CCCCCCGCGAGGAAGACCCCGGCAGTTGCGGGGGCGCCCCCCCTCGAGAG
GACGGGGGGAGCGACGAGGGCGACGTGTGCGCCGTGTGCACGGATGAGA
TCGCGCCCCACCTGCGCTGCGACACCTTCCCGTGCATGCACCGCTTCTGCA
TCCCGTGCATGAAAACCTGGATGCAATTGCGCAACACCTGCCCGCTGTGCA
ACGCCAAGCTGGTGTACCTGATAGTGGGCGTGACGCCCAGCGGGTCGTTC
AGCACCATCCCGATCGTGAACGACCCCCAGACCCGCATGGAGGCCGAGGA
GGCCGTCAGGGCGGGCACGGCCGTGGACTTTATCTGGACGGGCAATCAGC
GGTTCGCCCCGCGGTACCTGACCCTGGGGGGGCACACGGTGAGGGCCCTG
TCGCCCACCCACCCTGAGCCCACCACGGACGAGGATGACGACGACCTGGA
CGACGGTGAGGCGGGGGGCGGCGAGGACCCTGGGGGAGGAGGAGGAGG
GGGGGGGGGAGGGAGGAGTAGGCGGGCGGGCGGGCGAGGAAAGGGCGG
GCCGGGGAGGGGGCGTAACCTGATCGCGCCCCCCGTTGTCTCTTGCAGCA
GACTACGTACCGCCCGCCCCCCGCCGGACGCCCCGCGCCCCCCCACGCAG
AGGCGCCGCCGCGCCCCCCGTGACGGGCGGGGCGTCTCACGCAGCCCCCC
AGCCGGCCGCGGCTCGGACAGCGCCCCCCTCGGCGCCCATCGGGCCACAC
GGCAGCAGTAACACTAACACCACCACCACCAACAGCAGCGGCGGCGGCTC
CCGCCAGTCGCGAGCCGCGGTGCCGCGGGGGGCGTCTGGCCCCTCCGGGG
GGGTTGGGGTGGGGGTTGGGGTTGTTGAAGCGGAGGCGGGGCGGCCGAG
GGGCCGGACGGGCCCCCTTGTCAACAGACCCGCCCCCCTTGCAAACAACA
GAGACCCCATAGTGATCAGCGACTCCCCCCCGGCCTCTCCCCACAGGCCCC

CCGCGGCGCCCATGCCAGGCTCCGCCCCCCGCCCCGGTCCCCCCGCGTCCG
CGGCCGCGTCGGGCCCGCGCGCCCCCGCGCGGCCGTGGCCCCGTGCGTGC
GAGCGCCGCCTCCGGGGCCCGGCCCCCGCGCCCCGGCCCCCGGGGCGGAG
CCGGCCGCCCGCCCCGCGGACGCGCGCCGTGTGCCCCAGTCGCACTCGTC
CCTGGCTCAGGCCACGAACCAAGAACAGAGTCTGTGCCGGGCGCGTGCGA
CGGTGGCGCGCGGCTCGGGGGGGCCGGGCGTGGAGGGTGGACACGGGCC
CTCCCGCGGCGCCGCCCCCTCCGGCGCCCCCCCGCTCCCCTCCGCCGCCTC
TGTCGAGCAGGAGGCGGCGGTGCGTCCGAGGAAGAGGCGCGGGTCGGGC
CAGGAAAACCCCTCCCCCCAGTCCACGCGTCCCCCCCTCGCGCCGGCAGG
GGCCAAGAGGGCGGCGACGCACCCCCCCTCCGACTCAGGGCCGGGGGG
CGCGGCCAGGGTGGGCCCGGGACCCCCCTGACGTCCTCGGTGGCCTCCGC
CTCTTCCTCCTCCGCCTCTTCCTCCTCGGCCCCGACTCCCGCGGGGGCCGC
CTCTTCCGCCACCGGGGCCGCGTCCTCCTCCGCTTCCGCCTCCTCGGGCGG
GGCCGTCGGTGCCCTGGGAGGGAGACAAGAGGAAACCTCCCTCGGCCCCC
GCGCTGCTTCTGGGCCGCGGGGGCCGAGGAAGTGTGCCCGGAAGACGCG
CCACGCGGAGACTTCCGGGGCCGTCCCCGCGGGCGGCCTCACGCGCTACC
TGCCCATCTCGGGGGTCTCTAGCGTGGTCGCCCTGTCGCCTTACGTGAACA
AGACGATCACGGGGGACTGCCTGCCCATCCTGGACATGGAGACGGGGAAC
ATCGGGGCGTACGTGGTCCTGGTGGACCAGACGGGAAACATGGCGACCCG
GCTGCGGGCCGCGGTCCCCGGCTGGAGCCGCCGCACCCTGCTCCCCGAGA
CCGCGGGTAACCACGTGACGCCCCCCGAGTACCCGACGGCCCCCGCGTCG
GAGTGGAACAGCCTCTGGATGACCCCCGTGGGGAACATGCTGTTCGACCA
GGGCACCCTAGTGGGCGCCCTGGACTTCCGCAGCCTGCGGTCTCGGCACC
CGTGGTCCGGGGAGCAGGGGGCGTCGACCCGGGACGAGGGAAAACAATA
AGGGACGCCCCCCGTGTTTGTGGGGAGGGGGGGGGTCGGGCGCTGGGT
GGTCTCTGGCCGCGCCCACTACACCAGCCAATCCGTGTCGGGGAGGTGGA
AAGTGAAAGACACGGGCACCACACACCAGCGGGTCTTTTGTGTTGGCCCT
AATAAAAAAAAAACTCAGGGGATTTTGCTGTCTGTTGGGAAATAAAGGTTT
ACTTTTGTATCTTTTCCCTGTCTGTGTTGGATGTATCGCGGGGGTGCGTGGG
AGTGGGGGTGCGTGGGAGTGGGGGTGCGTGGGAGTGGGGGTGCGTGGGA
GTGGGGGTGCGTGGGAGTGGGGGTGCGTGGGAGTGGGGGTGCGTGGGAG
TGGGGGTGCGTGGGAGTGGGGGTGCGTGGGAGTGGGGGTGCCATGTTGGG
CAGGCTCTGGTGTTAACCACAGAGCCGCGGCCCGGGCTGCCTGACCACCG
ATCCCCGAAAGCATCCTGCCACTGGCATGGAGCCAGAACCACAGTGGGTT
GGGTGTGGGTGTTAAGTTTCCGCGAGCGCCTGCCCGCCCGGACTGACCTG
GCCTCTGGCCGCCACAAAGGGCGGGGGGGGTTAACTACACTATAGGGCAA
CAAAGGATGGGAGGGGTAGCGGGGCGGGACGGGGCGCCCAAAAGGGGGT
CGGCCACACCACAGACGTGGGTGTTGGGGGGTGGGGGGAGGGGTGGGGG
GGGGGGAAGACAGAAACAGGAACATAGTTAGAAAACAAGAATGCGGTGC
AGCCAGAGAATCACAGGAGACGAGGGGATGGGCGTGTTGGTTACCAACCC
ACACCCAGGCATGCTCGGTGGTATGAAGGAGGGGGGGCGGTGCTTCTTAG
AGACCGCCGGGGGACGTGGGGTTGGTGTGCAAAGGCACGCGCACCCGCG
CCGGCCAGGTGGGCCGGTACCCCATCCCCCCTCCCCCGACCCTTCCCACCC
CCGCGTGCCAGAGATCACCCCGGTCCCCCGGCACCCGCCACTCCTCCATAT

CCTCGCTTTAGGAACAACTTTAGGGGGGGTACACACGCGCCGTGCATTTCC
TTCCACACCCCCCCTCCCCCGCACTCCCCCCCCCCAGGCAGTAAGACCCAA
GCATAGAGAGCCAGGCACAAAAACACAGGCGGGGTGGGACACATGCCTTC
TTGGAGTACGTGGGTCATTGGCGTGGGGGGGGGGGTTACAGCGACACCGG
CCGACCCCCTGGCGGTCTTCCAGCCGGCCCTTAGATAAGGGGGCAGTTGGT
GGTCGGACGGGTAAGTAACAGAGTCTGACTAAGGGTGGGAGGGGGGGAA
AAGAACGGGCTGGTGTGCTGTAACACGAGCCCACCCGCGAGTGGCGTGGC
CGACCTTAGCCTCTGGGGCGCCCCCTGTCGTTTGGGTCCCCCCCTCTATTGG
GGAGAAGCAGGTGTCTAACCTACCTGGAAACGCGGCGTCTTTGTTGAACG
ACACCGGGGCGCCCTCGACGAGTGGGATAACGGGGGAGGAAGGGAGGGA
GGAGGGTACTGGGGGTGAAGAAGGGGGGGGGGAGAAGCGAGAACAGGA
AAGGCGGTGGAGCCCGGCAGAACACCGAGGAAAAAAAAAAACCACAGC
GCATGCGCCGGGCCGTTGTGGGGCCCCGGGCCGGGGCCCCTTGGGTCCGC
CGGGGCCCCGGGCCGGGCCGCCACGGGGGCCGGCCGTTGGCGGTAACCCC
GAGTGTTCATCTCAGGCCCCGGGCCGGGAACCCGGAAAAGCCTCCGGGGG
GCCTTTTTCGCGTCGCGTGCCGGCGAGCGGGTCCGGACGGGGCCCGGACC
GCCGCGGTCGGGGGCCCCTCGTCCCGGGCCGTACGCGGCCTTCGCCCCGT
GAGGGGACAGACGAACGAAACATTCCGGCGACGGAACGAAAAACACCCC
AGACGGGTTAAAGAAACAGAAACCGCAACCCCCACCACCCCCGAAACGG
GGAAAACGAAAAAACAGACCAGCGGCCGGCCGGCGCTTAGGGGGAGGAT
GTCGCCGACGCCCCTTGGCCGCCCCGGCTGCAGGGGGGCCCGGAGAGCCG
CGGCACCCGGACGCGCCCGGAAAGTCTTTCGCACCACCGGCGA.

supplementary results 2

New strain HSV-1-LXMW LAT2 From 118783 to 127151bp

TATAAAAGCGGGGGGCGCGGCCGTGCCGATCGCCGGTGGTGCGAAAGACT
TTCCGGGCGCGTCCGGGTGCCGCGGCTCTCCGGGCCCCCCTGCAGCCGGG
GCGGCCAAGGGGCGTCGGCGACATCCTCCCCCTAAGCGCCGGCCGGCCGC
TGGTCTGTTTTTTCGTTTTCCCCGTTTCGGGGGTGGTGGGGGTTGCGGTTTC
TGTTTCTTTAACCCGTCTGGGGTGTTTTTCGTTCCGTCGCCGGAATGTTTCG
TTCGTCTGTCCCCTCACGGGGCGAAGGCCGCGTACGGCCCGGGACGAGGG
GCCCCCGACCGCGGCGGTCCGGGCCCCGTCCGGACCCGCTCGCCGGCACG
CGACGCGAAAAAGGCCCCCCGGAGGCTTTTCCGGGTTCCCGGCCCGGGGC
CTGAGATGAACACTCGGGGTTACCGCCAACGGCCGGCCCCCGTGGCGGCC
CGGCCCGGGGCCCCGGCGGACCCAAGGGGCCCCGGCCCGGGGCCCCACA
ACGGCCCGGCGCATGCGCTGTGGTTTTTTTTTTTCCTCGGTGTTCTGCCGGG
CTCCACCGCCTTTCCTGTTCTCGCTTCTCCCCCCCCCCTTCTTCACCCCCAG
TACCCTCCTCCCTCCCTTCCTCCCCCGTTATCCCACTCGTCGAGGGCGCCCC
GGTGTCGTTCAACAAAGACGCCGCGTTTCCAGGTAGGTTAGACACCTGCTT
CTCCCCAATAGAGGGGGGGACCCAAACGACAGGGGGCGCCCCAGAGGCT
AAGGTCGGCCACGCCACTCGCGGGTGGGCTCGTGTTACAGCACACCAGCC
CGTTCTTTTCCCCCCCTCCCACCCTTAGTCAGACTCTGTTACTTACCCGTCC
GACCACCAACTGCCCCCTTATCTAAGGGCCGGCTGGAAGACCGCCAGGGG
GTCGGCCGGTGTCGCTGTAACCCCCCCCCCCACGCCAATGACCCACGTACT

CCAAGAAGGCATGTGTCCCACCCCGCCTGTGTTTTTGTGCCTGGCTCTCTAT
GCTTGGGTCTTACTGCCTGGGGGGGGGGAGTGCGGGGGAGGGGGGGTGTG
GAAGGAAATGCACGGCGCGTGTGTACCCCCCCTAAAGTTGTTCCTAAAGCG
AGGATATGGAGGAGTGGCGGGTGCCGGGGGACCGGGGTGATCTCTGGCAC
GCGGGGGTGGGAAGGGTCGGGGGAGGGGGGATGGGGTACCGGCCCACCT
GGCCGGCGCGGGTGCGCGTGCCTTTGCACACCAACCCCACGTCCCCCGGC
GGTCTCTAAGAAGCACCGCCCCCCCTCCTTCATACCACCGAGCATGCCTGG
GTGTGGGTTGGTAACCAACACGCCCATCCCCTCGTCTCCTGTGATTCTCTG
GCTGCACCGCATTCTTGTTTTCTAACTATGTTCCTGTTTCTGTCTTCCCCCCC
CCCCACCCCTCCGCCCCACCCCCCAACACCCACGTCTGTGGTGTGGCCGAC
CCCCTTTTGGGCGCCCCGTCCCGCCCCGCTACCCCTCCCATCCTTTGTTGCC
CTATAGTGTAGTTAACCCCCCCCGCCCTTTGTGGCGGCCAGAGGCCAGGTC
AGTCCGGGCGGGCAGGCGCTCGCGGAAACTTAACACCCACACCCAACCCA
CTGTGGTTCTGGCTCCATGCCAGTGGCAGGATGCTTTCGGGGATCGGTGGT
CAGGCAGCCCGGGCCGCGGCTCTGTGGTTAACACCAGAGCCTGCCCAACA
TGGCACCCCCACTCCCACGCACCCCCACTCCCACGCACCCCCACTCCCACG
CACCCCCACTCCCACGCACCCCCACTCCCACGCACCCCCACTCCCACGCAC
CCCCACTCCCACGCACCCCCACTCCCACGCACCCCCACTCCCACGCACCCC
CGCGATACATCCAACACAGACAGGGAAAAGATACAAAAGTAAACCTTTATT
TCCCAACAGACAGCAAAATCCCCTGAGTTTTTTTTTTTATTAGGGCCAACA
CAAAAGACCCGCTGGTGTGTGGTGCCCGTGTCTTTCACTTTCCACCTCCCC
GACACGGATTGGCTGGTGTAGTGGGCGCGGCCAGAGACCACCCAGCGCCC
GACCCCCCCCCCTCCCCACAAACACGGGGGGCGTCCCTTATTGTTTTCCCT
CGTCCCGGGTCGACGCCCCCTGCTCCCCGGACCACGGGTGCCGAGACCGC
AGGCTGCGGAAGTCCAGGGCGCCCACTAGGGTGCCCTGGTCGAACAGCAT
GTTCCCCACGGGGGTCATCCAGAGGCTGTTCCACTCCGACGCGGGGGCCG
TCGGGTACTCGGGGGGCGTCACGTGGTTACCCGCGGTCTCGGGGAGCAGG
GTGCGGCGGCTCCAGCCGGGGACCGCGGCCCGCAGCCGGGTCGCCATGTT
TCCCGTCTGGTCCACCAGGACCACGTACGCCCCGATGTTCCCCGTCTCCAT
GTCCAGGATGGGCAGGCAGTCCCCCGTGATCGTCTTGTTCACGTAAGGCGA
CAGGGCGACCACGCTAGAGACCCCCGAGATGGGCAGGTAGCGCGTGAGGC
CGCCCGCGGGGACGGCCCCGGAAGTCTCCGCGTGGCGCGTCTTCCGGGCA
CACTTCCTCGGCCCCCGCGGCCCAGAAGCAGCGCGGGGGCCGAGGGAGG
TTTCCTCTTGTCTCCCTCCCAGGGCACCGACGGCCCCGCCCGAGGAGGCG
GAAGCGGAGGAGGACGCGGCCCCGGTGGCGGAAGAGGCGGCCCCCGCGG
GAGTCGGGGCCGAGGAGGAAGAGGCGGAGGAGGAAGAGGCGGAGGCCA
CCGAGGACGTCAGGGGGGTCCCGGGCCCACCCTGGCCGCGCCCCCCCGGC
CCTGAGTCGGAGGGGGGGTGCGTCGCCGCCCTCTTGGCCCCTGCCGGCGC
GAGGGGGGGACGCGTGGACTGGGGGGAGGGGTTTTCCTGGCCCGACCCG
CGCCTCTTCCTCGGACGCACCGCCGCCTCCTGCTCGACAGAGGCGGCGGA
GGGGAGCGGGGGGGCGCCGGAGGGGGCGGCGCCGCGGGAGGGCCCGTGT
CCACCCTCCACGCCCGGCCCCCCCGAGCCGCGCGCCACCGTCGCACGCGC
CCGGCACAGACTCTGTTCTTGGTTCGTGGCCTGAGCCAGGGACGAGTGCG
ACTGGGGCACACGGCGCGCGTCCGCGGGGCGGGCGGCCGGCTCCGCCCC

GGGGGCCGGGGCGCGGGGGCCGGGCCCCGGAGGCGGCGCTCGCACGCAC
GGGGCCACGGCCGCGCGGGGGCGCGCGGGCCCGACGCGGCCGCGGACGC
GGGGGGACCGGGGCGGGGGGCGGAGCCTGGCATGGGCGCCGCGGGGGGC
CTGTGGGGAGAGGCCGGGGGGGAGTCGCTGATCACTATGGGGTCTCTGTT
GTTTGCAAGGGGGGCGGGTCTGTTGACAAGGGGGCCCGTCCGGCCCCTCG
GCCGCCCCGCCTCCGCTTCAACAACCCCAACCCCCACCCCAACCCCCCCG
GAGGGGCCAGACGCCCCCCGCGGCACCGCGGCTCGCGACTGGCGGGAGC
CGCCGCCGCTGCTGTTGGTGGTGGTGGTGTTAGTGTTACTGCTGCCGTGTG
GCCCGATGGGCGCCGAGGGGGGCGCTGTCCGAGCCGCGGCCGGCTGGGG
GGCTGCGTGAGACGCCCCGCCCGTCACGGGGGGCGCGGCGGCGCCTCTGC
GTGGGGGGGCGCGGGGCGTCCGGCGGGGGGCGGGCGGTACGTAGTCTGCT
GCAAGAGACAACGGGGGGCGCGATCAGGTTACGCCCCCTCCCCGGCCCGC
CCTTTCCTCGCCCGCCCGCCCGCCTACTCCTCCCTCCCCCCCCCCCTCCTCC
TCCTCCCCCAGGGTCCTCGCCGCCCCCCGCCTCACCGTCGTCCAGGTCGTC
GTCATCCTCGTCCGTGGTGGGCTCAGGGTGGGTGGGCGACAGGGCCCTCA
CCGTGTGCCCCCCCAGGGTCAGGTACCGCGGGGCGAACCGCTGATTGCCC
GTCCAGATAAAGTCCACGGCCGTGCCCGCCCTGACGGCCTCCTCGGCCTCC
ATGCGGGTCTGGGGGTCGTTCACGATCGGGATGGTGCTGAACGACCCGCT
GGGCGTCACGCCCACTATCAGGTACACCAGCTTGGCGTTGCACAGCGGGC
AGGTGTTGCGCAATTGCATCCAGGTTTTCATGCACGGGATGCAGAAGCGGT
GCATGCACGGGAAGGTGTCGCAGCGCAGGTGGGGCGCGATCTCATCCGTG
CACACGGCGCACACGTCGCCCTCGTCGCTCCCCCCGTCCTCTCGAGGGGG
GGCGCCCCCGCAACTGCCGGGGTCTTCCTCGCGGGGGGGGCTCCCCCCCG
AGACCGCCCCCCCATCCACGCCCTGCGGCCCCAGCAGCCCCGTCTCGAAC
AGTTCCGTGTCCGTGCTGTCCGCCTCGGAGGCGGAGTCGTCGTCATGGTGG
TCGGCGTCCCCCCGCCCCCCCACTTCGGTCTCCGCCTCCGAGTCGCTGCTG
TCCGGCAGGTCTCGGTCGCAGGGAAACACCCAGACATCCGGGGCGGGCTA
AGGGGAAAAAAGGGGGGGTGGGTAAGAATGGGGGGGATTTCCCGCGTCA
ATCAGCGCCCACGAGTTCCCCCTCCCCCCCGCCTCACAAAGTCCTGCCCCC
CTGCTGGCCTCGGAAGAGGGGGGAGAAAGGGGTCTGCAACCAAAGGTGG
TCTGGGTCCGTCCTTTGGATCCCGACCCCTCTTCTTCCCTCTTCTCCCGCCC
TCCAGACGCACCGGAGTCGGGGGTCCCACGGCGTCCCCCAAATATGGCGG
GCGGCTCCTCCCCACCCCCCTAGATGCGTGTGACTAAGGGGGCCCTGCGTG
TGAGTCAGTGGGACCACGCCCCCTAACACGGCGACCCCGGTCCCTGTGTG
TTTGTTGTGGGGGCGTGTCTCTGTGTATGAGTCAGGGGGGGGGGGTCCCAC
GGCGACCCCGGGCCCTGCGTCTGAGTCAAAGGGGCCATGTGTATGTGTTGG
GGGTCTGTATATATAAAGTCAGGGGGTCACATGGCGACCCCTAACAGGGCG
ACCCCGGTCCCTGTATATATAGGGTCAGGGGGTTCCGCGCCCCCTAACATGG
CGCCCCCGGTCCCTGTATATATAGTGTCACGGGGTTCCACGCCCCCTAACAT
GGCGCCCCAACATGGCGCCCGGCTCCCGTGTATGAGTGGGGGTCCCCCAA
CATGGCGGCCGGTTCCAGTGTAAGGGTCGGGGGTCCCCCAACATGGCGCC
CCCCAATATGGCGCCCCCCAATATGGCGCCCCAGACATGGCGCCCGGCCCC
TCACCTCGCGCTGGGGGCGGCCCTCAGGCCGGCGGGTACTCGCTCCGGGG
CGGGGCTCCATGGGGGTCGTATGCGGCTGGAGGGTCGCGGACGGAGGGTC

CCTGGGGGTCGCAACGTAGGCGGGGCTTCTGTGGTGATGCGGAGAGGGGG
CGGCCCGAGTCTGCCTGGCTGCTGCGTCTCGCTCCGAGTGCCGAGGTGCA
AATGCGACCAGACCGTCGGGCCAGGGCTAACTTATACCCCACGCCTTTCCC
CTCCCCAAAGGGGCGGCAGTGACGATTCCCCCAATGGCCGCGCGTCCCAG
GGGAGGCAGGCCCACCGCGGGGCGGCCCCGTCCCCGGGGACCAACCCGG
CGCCCCCAAAGAATATCATTAGCATGCACGGCCCGGCCCCCGATTTGGGGG
ACCAACCCGGTGTCCCCCAAAGAACCCCATTAGCATGCCCCTCCCGCCGAC
GCAACAGGGGCTTGGCCTGCGTCGGTGCCCCGGGGCTTCCCGCCTTCCCG
AAGAAACTCATTACCATACCCGGAACCCCAGGGGACCAATGCGGGTTCATT
GAGCGACCCGCGGGCCAATGCGCGAGGGGCCGTGTGTTCCGCCAAAAAA
GCAATTAACATAACCCGGAACCCCAGGGGAGTGGTTACGCGCGGCGCGGG
AGGCGGGGAATACCGGGGTTGCCCATTAAGGGCCGCGGGAATTGCCGGAA
GCGGGAAGGGCGGCCGGGGCCGCCCATTAATGAGTTTCTAATTACCATACC
GGGAAGCGGAACAAGGCCTCTGCAAGTTTTTAATTACCATACCGGGAAGTG
GGCGGCCCGGCCCACTGGGCGGGAGTTACCGCCCAGTGGGCCGGGCCCCA
ACGACTCGGTGGACGCTGGTTGGCCGGGCCCCGCCGCGCTGGCGGCCGCC
GATTGGCCAGTCCCGCCCTCCGAGGGCGGGCCCGCCTCGGGGGCGGGCCG
GCTCCAAGCGTATATATGCGCGGCTCCTGCCATCGTCTCTCCGGAGAGCGG
CTTGGTGCGGAGCTCCCGGGAGCTCCGCGGAAGACCCAGGCCGCCTCGGG
TGTAACGTTAGACCGAGTTCGCCGGGCCGGCTCCGCGGGCCAGGGCCCGG
GCCCGGGCACGGGCCTCGGGCCCCAGGCACGGCCCGATGACCGCCTCGGC
CTCCGCCACCCGGCGCCGGAACCGAGCCCGGTCGGCCCGCTCGCGGGCCC
ACGAGCCGCGGCGCGCCAGGCGGGCGGCCGAGGCCCAGACCACCAGGTG
GCGCACCCGGACGTGGGGCGAGAAGCGCACCCGCGTGGGGGTCGCGGGG
GTCGCGGGGGTCGCGGGGGTCGCGGGGGTCGCGGGGGGCTCCGGCGCCC
CCTCCCCGCCCGCGCGTCGCAGGCGCAGGCGCGCCAGGTGCTCTGCGGTG
ACGCGCAGGCGGAGGGCGAGGCGCGGCGGAAGGCGGAAGGGGCGTGAG
GGGGGGTGGGAGGGGTTAGCCCCGCCCCCCGGGCCCGCGCCGGGCGGTG
GGGACCGGGGGCGGGGGGCGGCGGCGGTGGGCCGGGCCTCTGGCGCCGG
CTCGGGCGGGGGGCTGTCCGGCCAGTCGTCGTCGTCGTCGTCGTCGTCGG
ACGCGGACTCGGGAACGTGGAGCCACTGGCGCAGCAGCAGCGAACAAGA
AGGCGGGGGCCCACTGGCGGGGGGCGGCGGCGGGGCGGCCGCGGGCGCG
CTCCTGACCACGGGTTCCGAGTTGGGCGTGGAGGTTACCTGGGACTGTGC
GGTTGGGACCGCGCCCGTGGGCCCGGGCGGCCGGGGGCGGCGGGGGCCG
CGATGGCGGCGGCGGCGGGCCATGGAGACAGAGAGCGTGCCGGGGTGGT
AGAGTTTGACAGGCAAGCATGTGCGTGCAGAGGCGAGTAGTGCTTGCCTG
TCTAACTCGCTAGTCTCGGCCGCGGGGGGCCCGGGCTGCCCGCCGCCGCG
CTTTAAAGGGCCGCGCGCGACCCCCGGGGGGTGTGTTTTGGGGGGGGGCC
CGTTTTCGGGGTCTGGCCGCTCCTCCCCCCGCTCCTCCCCCCGCTCCTCCCC
CCGCTCCTCCCCCCGCTCCTCCCCCCGCTCCTCCCCCCGCTCCTCCCCCCGC
TCCTCCCCCCGCTCCTCCCCCCGCTCCTCCCCCCGCTCCTCCCCCCGCTCCT
CCCCCCGCTCCTCCCCCCGCTCCTCCCCCCGCTCCTCCCCCCGCTCCTCCCC
CCGCTCCTCCCCCCGCTCCTCCCCCCGCTCCCGCGGCCCCGCCCCCCAACG
CCCGCCGCGCGCGCGCACGCCGCCCGGACCGCCGCCCGCCTTTTTGCGC

GCGCGCGCGCCCGCGGGGGGCCCGGGCTGCCACAGGTGAAACAACGCCA
ACAGAGCACGGCGCACTCCGCACGTCACACGTCACGTCATCCACCACACC
TGCCCACCAACACAACTCACAGCGACAACTCACCGCGCAACAACTCCTGT
TCCTCATCCACACGTCACCGCGCACCTCCCGCTCCTCCAGACGTACCCCGG
CGCAACACACCGCTCCTGCTACACACCACCGCCCCTCCCCAGCCCCAGCCC
TCCCCAGCCCCAGCCCTCCCCAGCCCCAGCCCTCCCCAGCCCCAGCCCTCC
CCAGCCCCAGCCCTCCCCAGCCCCAGCCCTCCCCAGCCCCAGCCCTCCCCA
GCCCCAGCCCTCCCCAGCCGCGTCCCGCGCTCCCTCGGGGGGGTTCGGGC
ATCTCTACCTCAGTGCCGCCAATCTCAGGTCAGAGATCCAAACCCTCCGGG
GGCGCCCGCGCACCACCACCGCCCCTCGCCCCCCCCCCCCCCCCCCCCCC
CCCCCCCCCCCCCCCCCCCCCCCCCCCCCCCCCCCCCCCCCGCCCCCCCC
CCCCCGCCCCCCCCGCCCCTCCCCCCCCCCCCCCCCCCCCCCCCCCGCCC
CCCCCGCCCCTCCCCCCCCCCCCCCCCCCCCCCGCCCCTCGCCCCCTCCCG
CCCCCCCCCCCCCCCCCCGCCCCCCCCCCCCCCCCCCTCCCGCCCCTCGCC
CCCTCCCGCCCCTCGCCCCCTCCCGCCCCTCGCCCCCTCCCGCCCCTCGAAT
AAACAACGCTACTGCA.

supplementary results 3
HSV-1-LXMW LAP1 (7589-9589bp)
TCGGCACGGCCGCGCCCCCGCTTTTATAAAGGCTCAGATGACGCAGCAAA
AACAGGCCACAGCACCACGTGGGTAGGTGATGTAATTTTATTTTCCTCGTCT
GCGGCCTAATGGATTTCCGGGCGCGGTGCCCCTGTCTGCAGAGCACTTAAC
GGATTGATATCTCGCGGGCACGCGCGCCCTTAATGGACCGGCGCGGGGCGG
GGGGCCGGATACCCACACGGGCGGGGGGGGGGTGTCGCGGGCCGTCTGCT
GGCCCGCGGCCACATAAACAATGACTCGGGGCCTTTCTGCCTCTGCCGCTT
GTGTGTGCGCGCGCCGGCTCTGCGGTGTCGGCGGCGGCGGCGGTGGCCGC
CGTGTTCGGTCTCGGTAGCCGGCCGGCGGGTGGACTCGCGGGGGGCCGGA
GGGTGGAAGGCAGGGGGGTGTAGGATGGGTATCAGGACTTCCACTTCCCG
TCCTTCCATCCCCCGTTCCCCTCGGTTGTTCCTCGCCTCCCCCAACACCCCG
CCGCTTTCCGTTGGGGTTGTTATTGTTGTCGGGATCGTGCGGGCCGGGGT
CGCCGGGGCAGGGGCGGGGGCGTGGGCGGGGGTGCTCGTCGATCGACCG
GGCTCAGTGGGGGCGTGGGGTGGGTGGGAAAAGGCGAGGAGACTGGGGT
GGGGGGTGTCGGTGGGTGGTTGTTTTTTGTGGTTGTTTTTTGTGTCTGTTCC
CGTCCCCCGTCACCCCCCTCCCTCCGTCCCCTCCGTCCCCCCGTCGCGGGT
GTTTGTGTTTGTTTATTCCGACATTGGTTTGTTTAAATAAACACAGCCGTTCT
GCGTGTCTGTTCTTGCGTGTGGCTGGGGGCTTATATGTGGGGTCCCGGGGG
CGGGATGGGGTTTAGCGGCGGGGGGCGGCGCGCCGGACGGGGCGCTGGA
GATAACGGCCCCCGGGGAACGGGGGACCGGGGCTGGGTCTCCCGAGGTGG
GTGGGTGGGCGGCGGTGGCCGGGCCGGGCCGGGCCGGGCCGGGCTGGGT
GGGCGGGGTTTGGAAAAACGAGGAGGCGGAGGAGGAGGGGGGGAGACG
GGGGGAAAGCAAGGACACGGCCCGGGGGGTGGGAGCGCGGGCCGGGCC
GCTCGTAAGAGCCGCGACCCGGCCGCCGGGGAGCGTTGTCGCCGTCGGTC
TGCCGGCCCCCGTCCCTCCCTTTTTTGACCAACCAGCGCCCCCCCCCTCAC
CACCATTCCTACTACCACCACCACCACCACCACCGACACCTCCCGCGCACC

CCCGCCCACATCCCCCCAACCCGCACCACGAGCACGGGTTGGGGGTAGCA
GGGGATCAAAGGGGGACAAAGCCGGCGGGGCGGTTCGGGGGGGGGGGG
GGGGGCGGGAGACCGAGTAGGCCCGCCCATCCGCGGCCCCTCCCGGCAGC
CACGCCCCCAGCGTCGGGTGTCACGGGGAAAGAGCAGGGGAGAGGGGAG
AGGGGGGGAGAGGGGAGAGGGGGGGAGAGGGGAGAGGGGGGGAGAGG
GGAGAGGGGGGGAGAGGGGAGAGGGGGGGAGAGGGGAGAGGGGGGGA
GAGGGGAGAGGGGGGGAGAGGGGAGAGGGGGGGAGAGGGGAGAGGGG
GGGAGAGGGGGGGAGAGGGGAGAGGGGGTATATAAACCAACGAAAAGCG
CGGGAACGGGGATACGGGGCTTGTGTGGCACGACGTAGTGGTTGTGTTACT
GGGCAAACACTTGGGGACTGTAGGTTTCTGTGGGTGCCGACCCTAGGCGCT
ATGGGGATTTTGGGTTGGGTCGGGCTTATTGTCGTTGGGGTTTTGTGTGTGC
GGGGGGGCTTGCCTTCAACCGAATATGTTATTCGGAGTCGGGTGGCTCGAG
AGGTGGGGGATATATTAAAGGTGCCTTGTGTGCCGCTCCCGTCTGACGATCT
TGATTGGCGTTACGAGACCCCCTCGGCTATAAACTATGCTTTGATAGACGGT
ATATTTTTGCGTTATCACTGTCCCGGATTGGACACGGTCTTGTG.

supplementary results 4

HSV-1-LXMW LAP2 (116797-118797bp)

GTGGAGGTGGGTCCGTGGGGGTGTCCAGATCAATAACGACAAACGGCCCC
TCGTTCCTACCAGACAAGCTATCGTAGGGGGGCGGGGGATCAGCAAACGC
GTTCCCCGCGCTCCATAGACCCGCGTCGGGTTGCGCCGCCTCCGAAGCCAT
GGATGCGCCCCAAAGCCACGACTCCCGCGCGCTAGGTCCTTGGGGTAAGG
GAAAAGGCCCTACTCCCCATCCAAGCCAGCCAAGTTAACGGGCTACGCCTT
CGGGAATGGGACTGGCACCCCGGCGGATTTTGTTGGGCTGGCATGCGTCGC
CCAACCGAGGGCCGCGTCCACGGGACGCGCCTTTTATAACCCCGGGGGTC
ATTCCCAACGATCACATGCAATCTAACTGGCTCCCCTCTCCCCCCCTCTCCC
CTCTCCCCCCCTCTCCCCTCTCCCCCCCTCTCCCCTCTCCCCCCCTCTCCCC
TCTCCCCCCCTCTCCCCTCTCCCCCCCTCTCCCCTCTCCCCCCCTCTCCCCT
CTCCCCCCCTCTCCCCTCTCCCCCCCTCTCCCCTCTCCCCCCCTCTCCCCTC
TCCCCTCTGCTCTTTCCCCGTGACACCCGACGCTGGGGGCGTGGCTGCCGG
GAGGGGCCGCGGATGGGCGGGCCTACTCGGTCTCCCGCCCCCCCCCCCCC
CCCCCGAACCGCCCCGCCGGCTTTGTCCCCCTTTGATCCCCTGCTACCCCC
AACCCGTGCTCGTGGTGCGGGTTGGGGGGATGTGGGCGGGGGTGCGCGGG
AGGTGTCGGTGGTGGTGGTGGTGGTGGTAGTAGGAATGGTGGTGAGGGGG
GGGGCGCTGGTTGGTCAAAAAAGGGAGGGACGGGGGCCGGCAGACCGAC
GGCGACAACGCTCCCCGGCGGCCGGGTCGCGGCTCTTACGAGCGGCCCGG
CCCGCGCTCCCACCCCCCGGGCCGTGTCCTTGCTTTCCCCCCGTCTCCCCC
CCCCTCCTCCTCCGCCTCCTCGTTTTTCCAAACCCCGCCCACCCAGCCCGG
CCCGGCCCGGCCCGGCCCGGCCACCGCCGCCCACCCACCCACCTCGGGAG
ACCCAGCCCCGGTCCCCCGTTCCCCGGGGGCCGTTATCTCCAGCGCCCCGT
CCGGCGCGCCGCCCCCCGCCGCTAAACCCCATCCCGCCCCCGGGACCCCA
CATATAAGCCCCCAGCCACACGCAAGAACAGACACGCAGAACGGCTGTGT
TTATTTAAACAAACCAATGTCGGAATAAACAAACACAAACACCCGCGACG
GGGGGACGGAGGGGACGGAGGGAGGGGGGTGACGGGGGACGGGAACAG

ACACAAAAAACAACCACAAAAAACAACCACCCACCGACACCCCCCACCC
CAGTCTCCTCGCCTTTTCCCACCCACCCCACGCCCCCACTGAGCCCGGTCG
ATCGACGAGCACCCCCGCCCACGCCCCCGCCCCTGCCCCGGCGACCCCCG
GCCCGCACGATCCCGACAACAATAACAACCCCAACGGAAAGCGGCGGGGT
GTTGGGGGAGGCGAGGAACAACCGAGGGGAACGGGGGATGGAAGGACG
GGAAGTGGAAGTCCTGATACCCATCCTACACCCCCCTGCCTTCCACCCTCC
GGCCCCCCGCGAGTCCACCCGCCGGCCGGCTACCGAGACCGAACACGGCG
GCCACCGCCGCCGCCGCCGACACCGCAGAGCCGGCGCGCACACACAA
GCGGCAGAGGCAGAAAGGCCCCGAGTCATTGTTTATGTGGCCGCGGGCCA
GCAGACGGCCCGCGACACCCCCCCCCGCCCGTGTGGGTATCCGGCCCCC
CGCCCCGCGCCGGTCCATTAAGGGCGCGCGTGCCCGCGAGATATCAATCCG
TTAAGTGCTCTGCAGACAGGGGCACCGCGCCCGGAAATCCATTAGGCCGC
AGACGAGGAAAATAAAATTACATCACCTACCCACGTGGTGCTGTGGCCTGT
TTTTGCTGCGTCATCTGAGCCTT
